# Agent-based simulations reveal the possibility of multiple rapid northern routes for the second Neanderthal dispersal from Western to Eastern Eurasia

**DOI:** 10.1371/journal.pone.0325693

**Published:** 2025-06-09

**Authors:** Emily Coco, Radu Iovita

**Affiliations:** 1 Interdisciplinary Center for Archaeology and the Evolution of Human Behavior (ICArEHB), Universidade do Algarve, Faro, Portugal; 2 Center for the Study of Human Origins, Department of Anthropology, New York University, New York, New York, United States of America; 3 Department of Anthropology, Yale University, New Haven, Connecticut, United States of America; 4 Department of Early Prehistory and Quaternary Ecology, University of Tübingen, Tübingen, Germany; Universita degli Studi di Ferrara, ITALY

## Abstract

Genetic and archaeological evidence imply a second major movement of Neanderthals from Western to Central and Eastern Eurasia sometime in the Late Pleistocene. The genetic data suggest a date of 120−80 ka for the dispersal and the archaeological record provides an earliest date of arrival in the Altai by ca. 60 ka. Because the number of archaeological sites linking the two regions is very small, the exact route taken and its timing have been the matter of considerable debate. In particular, climate change in this period modified landscapes considerably, changing the cost of moving in different directions. Here, we apply agent-based least-cost path simulations for the first time to Neanderthals, showing that they most likely took a northern route through the Urals and southern Siberia under all climate scenarios. Agents leaving either the southern or the northern Caucasus Mountains reach the Altai in less than 2000 years during two time windows when the climate was mild, in MIS 5e (the Last Interglacial) and in MIS 3. The latter coincides with the dated presence of Neanderthals at Chagyrskaya and Okladnikov Caves in the Altai. The results of this modeling approach demonstrate a remarkable east-west geographic connectivity of northern Eurasia via river corridors despite the presumed barriers of the Ural Mountains and major north-south flowing rivers. Our results highlight the unique strengths of agent-based simulations to reconstruct pathways for ancient migrations.

## Introduction

The advances during the last few decades of research have revealed that Neanderthals colonized a vast territory that stretched from Spain to Siberia [1–3]. The earliest Neanderthals likely arrived in Asia shortly before marine isotope stage (MIS) 6 (190−130 ka) [4,5]. Following MIS 6, we see a new, and genetically distinct, connection between European and Siberian Neanderthals dated between 120−80 ka during MIS 5; first documented in the genetic similarities between the Mezmaiskaya and Chagyrskaya fossils [2], this genetic evidence of a second movement of Neanderthals east now is confirmed by ancient DNA from sediments at several caves in Europe and Siberia [3]. Archaeologically, this genetic connection is accompanied by a number of sites in the Russian Plain, the Caucasus, and Siberia that share morphological characteristics in their stone tool assemblages belonging to the Middle Palaeolithic [6].

**Fig 1:**
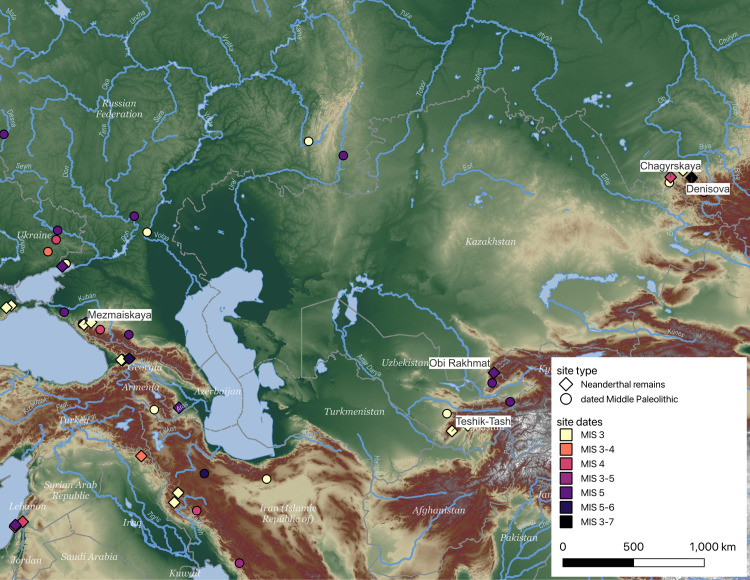
Current state of archaeological record for MIS 6 to MIS 3 in Eastern Eurasia between the Caucasus Mountains and the Altai Mountains. Labels provided for some sites with Neanderthal remains to orient the viewer. Base map is derived from a digital elevation model (SRTM15 + V2.5.5) provided by Open Topography [14] with hydrological features from Natural Earth.

Ancient human populations in Eurasia have consistently been structured by geographic changes. During the penultimate (MIS 12) and antepenultimate (MIS 6) glacial periods, continental ice sheets reached down to the Dnieper basin, almost completely separating Europe from Asia [7]. The earlier of the two may have contributed to the separation of Neanderthals (in the west) and their closely related Denisovan cousins, in eastern Asia [8]. The MIS 6 glacial expansion likely separated Siberian Neanderthals from European Neanderthals until glaciers retreated, reopening the connection between the Russian and West Siberian plains. It is unclear if the two populations came into contact during the succeeding warm interglacial (MIS 5e) or not, because there are too few sites (see Fig 1) [for example, as demonstrated by the ROCEEH Out of Africa Database, 9]. The next connection between the European and Siberian Neanderthals is documented solely through genetic evidence that suggests a dispersal of Neanderthals east at the end of MIS 5e and during the next cooling phase (MIS 5d-a, 110−70 ka) [3,10]. The colder phase that follows, MIS 4, is believed to have transformed the open Eurasian landscapes into a cold desert, with populations retreating into mountain refugia, where they persisted until the next warm phase, MIS 3. Broadly, the archaeological record of MIS 3 is characterized by an increased diversification and regionalization of stone tool assemblages [11], as well as by sparse, fragmented populations [12] occupying smaller and smaller territories [13].

Despite the genetic evidence for multiple long-distance dispersals by Neanderthals across Eurasia, we still have limited knowledge about the possible routes they would have taken to get to Siberia and the behavioral adaptations required for that journey. Archaeologists often use least-cost paths (LCP) to model past movement [15,16]. However, traditional LCP analyses make the assumption that the entire landscape is perfectly known in advance and that travelers will always choose the easiest and shortest route between two points [15]. This is not realistic for modeling movement in unknown environments. Here, we use an agent-based least cost path (AB-LCP) approach to more realistically model mobility decisions when a traveler can only use information about their local surroundings [17] and, importantly, when they do not have a specific destination they must reach. This methodology is similar to step selection models used to model animal movement and to investigate the peopling of Australia [18], but has yet to be applied to Neanderthals. Our model improves on traditional LCP analyses and simplifies the step selection methodology to reconstruct ancient migrations with as few assumptions as possible.

Here, we created a paleogeographically realistic agent-based least cost path model to simulate dispersal of Neanderthal populations between MIS 6 and MIS 3. Using cost surfaces derived from elevation, climate, hydrological, and glacier data, we explore possible paths that Neanderthals may have taken across Eurasia when dispersing out of the Caucasus Mountains.

## Materials and methods

### Agent-based least cost path model

We simulate agent-based cost-minimizing Lévy walks of mobile agents (ODD protocol of model available in S1 Appendix). The model is based on Gravel-Miguel and Wren’s agent-based least-cost path model [17]. This model was coded in NetLogo 6.3.0 [19] and is available at https://doi.org/10.17605/OSF.IO/H5BJK.

Agents traverse a landscape defined by a 1-km resolution raster of cost values (described below). To start, agents are placed on a random raster square in the Caucasus Mountains. At each tick of the model, the agent evaluates its local landscape by assessing all eight adjacent squares in a 360-degree radius and faces the square with the lowest cost value that had not been recently occupied. From there, the agent makes a number of steps determined by a Lévy walk probability equation [20–22]. Lévy walks are often used as a neutral model of hunter-gatherer mobility [21,23]; here, we use them to modulate between short- and long-distance movements with higher probabilities of short-distance movements that would have been more typical for everyday mobility. At each step of the Lévy walk, the agent evaluates the squares within a 100-degree radius and again moves to the adjacent square with the lowest cost value that had not been recently occupied; this is so the agent will not consider the squares behind itself to keep the agent moving in roughly the same direction for the entirety of the Lévy walk.

To simulate colonization behaviors, we allowed agents to move farther if they found themselves in “new territory” [24], meaning they had not recently occupied most of their surrounding grid squares. In this case, agents were allowed to double the number of steps they were taking as part of their Lévy walk. This behavior occurs infrequently, but allows for the possibility of long-distance colonization movements.

We run the model on cost rasters corresponding to MIS 6–3 (available at http://doi.org/10.17605/OSF.IO/AH2MK), a time span which bookends the estimated date of divergence of the Neanderthal populations represented by individuals at Mezmaiskaya (Caucasus) and Chagyrskaya (Altai) and the occupation dates of Chagyrskaya [2,3,6]. We varied the starting location of the agents, including areas north and south of the Caucasus Mountains (S1 Fig in S1 Appendix). These areas are derived from the locations of Middle Paleolithic sites in the region. Each iteration of the model is run for a total of 400,000 Lévy walks, which results in approximately 830,000 individual steps (SD = 392,309.7) on average across all modeled scenarios. We intentionally kept model runs short because we were interested in exploring the possibility of distinct long-distance dispersal routes [6], instead of simply calculating when general eastward population advance would eventually reach the Altai.

For each combination of isotope stage (MIS) and starting location in the Caucasus, we ran the model five times (S4 Table in S1 Appendix) to test for the potential of rapid long distance dispersals under our simple model assumptions. We identified multiple successful routes from this set of 110 total model runs.

### Building cost surfaces

The 1-km resolution cost rasters for this study were built using data from digital elevation models (DEMs) [14], paleoclimate data on annual precipitation [25], Pleistocene glacier extents [7], extents of modern rivers, lakes, and oceans [26–28], and paleogeographic data on Pleistocene glacier lake outbursts (S6 Table in S1 Appendix). Importantly, we do not include any information on the location of Middle Paleolithic sites when constructing our cost rasters, so agents do not consider them when moving.

We used a slope-dependent cost function to estimate energy expenditure by a walking individual via Llobera and Sluckin’s walker cost function [16,29]. The slope-based costs were used as a base map to which other costs were added. We increased the base cost of desert areas that received less than 250 mm of annual precipitation [30] to allow short movements across these regions [31,32]. We increase the cost for these cells by the amount equivalent to an increase from a slope of 0 degrees to a slope of 15 degrees [33–35], which is equivalent to a 74% increase in cost. We used glacier extent data from Batchelor et al. [7] to create glacial barriers. Similarly, we used modern lake and ocean extent data to define some of our water barriers. For rivers, we used average long-term water discard estimates to approximate river width [36,37]. Rivers with a width greater than a kilometer are considered barriers. Rivers with widths less than a kilometer were crossable, but given a 74%-increased cost [33–35].

In addition to modern lakes and rivers, we simulated paleohydrology using the Lake Flood function in QGIS [38]. We reviewed the literature for estimated Middle Pleistocene lake and sea levels (S7 Table in S1 Appendix) and used these values to "flood" the DEM based on estimated conditions for each marine isotope stage and substage (S8 Table in S1 Appendix). A full description of cost raster development can be found in the S1 Appendix. All derived rasters used for creating the cost rasters and the cost rasters themselves can be found at https://doi.org/10.17605/OSF.IO/H5BJK.

### Analysis of routes

Model outputs a series of XY coordinates documenting each step agents take in the model. We produced maps of paths taken for each climate scenario for both starting points (S2–9 Figs in S1 Appendix) by calculating the relative frequency with which agents occupied a given set of XY coordinates across all runs of each modeled scenario. We also identified successful individual runs by creating a 500 km buffer around the archaeological sites in the Altai and determining which paths had steps that fell within that buffer. Additionally, we calculated the Euclidean distance between the last set of coordinates for each model run and the 500 km buffer zone.

We were also interested in understanding the structure of landscape use during dispersals. Specifically, we investigated whether agents followed expectations of using landscape features like rivers to perform long-distance linear movements [24] and if there were certain areas agents occupied more redundantly. To look at linear movements, we calculated movement path angles and identified portions of the path where angle does not change for at least 5 steps in a row. For analyzing areas of redundant movement, we considered all model runs that moved beyond the Caucasus region, namely those during MIS 3, MIS 4, MIS 5a, MIS 5c, and MIS 5e. We calculated the frequency of agent occupation for each set of XY coordinates to identify those within 1 standard deviation of the mean occupation frequency.

Finally, we estimated arrival times to the Altai. To do so, we counted the number of steps, represented as XY coordinates, taken to reach the Altai during each of the successful model runs. We divided this number by the average annual distance traveled for hunter-gatherer populations occupying similar climates (S9 Table in S1 Appendix) [39,40].

## Results

### Route and timing of successful dispersals

**Fig 2:**
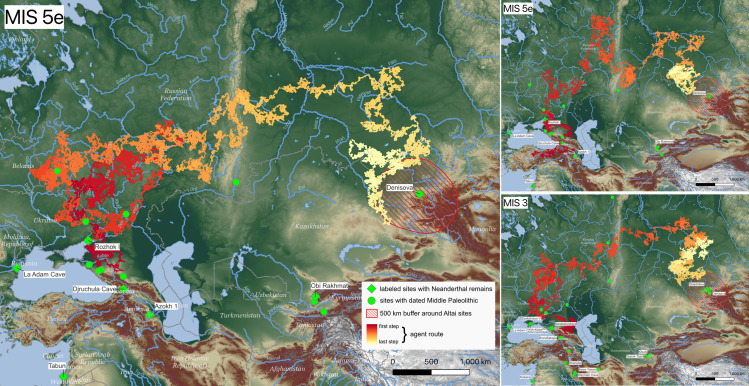
Geography and timing of successful routes that reach within 500 kilometers of the Altai site cluster. The modeled marine isotope stage that produced the route is displayed in the upper left corner of each map. Path color indicates step number with the start of the path in dark red and the end in light yellow. Sites with Neanderthal fossils are indicated with green diamonds; some are labeled. Other Middle Paleolithic sites are indicated with green circles. Base map is derived from a digital elevation model (SRTM15 + V2.5.5) provided by Open Topography [14] with hydrological features from Natural Earth.

We defined success as reaching a point within 500 km of the Altai site cluster. This is based on ethnographically-derived data demonstrating that mobile populations can move about 500 km within one year. Only three out of 110 runs were successful, all tracing an arc through the Urals and southern Siberia before reaching the Altai (see Fig 2). Two of the successful runs take place in MIS 5e and one during MIS 3. Additionally, two other unsuccessful runs reach within 1000 km of the Altai during MIS 5e and MIS 5c.

**Fig 3:**
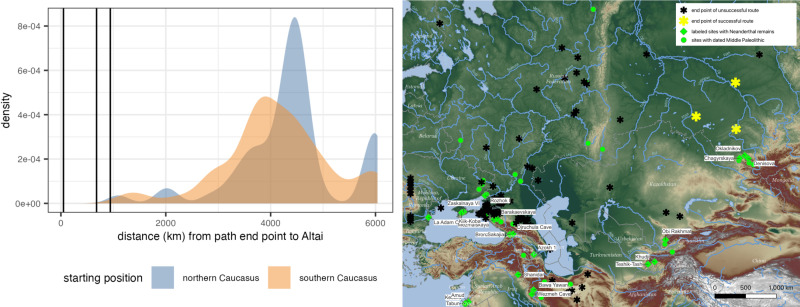
Locations of agent path end points. Density distribution of distance from end point of all modeled movement paths to a 500 km buffer around Altai site cluster (Denisova, Chagyrskaya, Okladnikov) (left). Distances from end points of successful paths to the buffer are indicated by the vertical black lines. Map of end points of all modeled movement paths (right). Endpoints of successful paths indicated in yellow and those of unsuccessful paths are indicated in black. Some unsuccessful paths terminate off the map. Base map of right panel is derived from a digital elevation model (SRTM15 + V2.5.5) provided by Open Topography [14] with hydrological features from Natural Earth.

The end points of most model runs are typically far away from the Altai cluster (see Fig 3). There are a few model scenarios where agents make it to the Ural Mountains, which is approximately halfway to the Altai; this occurs multiple times in MIS 5e, MIS 5c, MIS 4, and MIS 3 (S3, S5, S8, S9 Figs in S1 Appendix). These routes consistently take over a million steps to get halfway to the Altai, so it is possible that given more time these routes could end up in the Altai, but that outcome is not guaranteed nor is it clear how much more time would be required. Future iterations of this model may demonstrate the possibility of less rapid dispersals in additional time periods.

**Fig 4:**
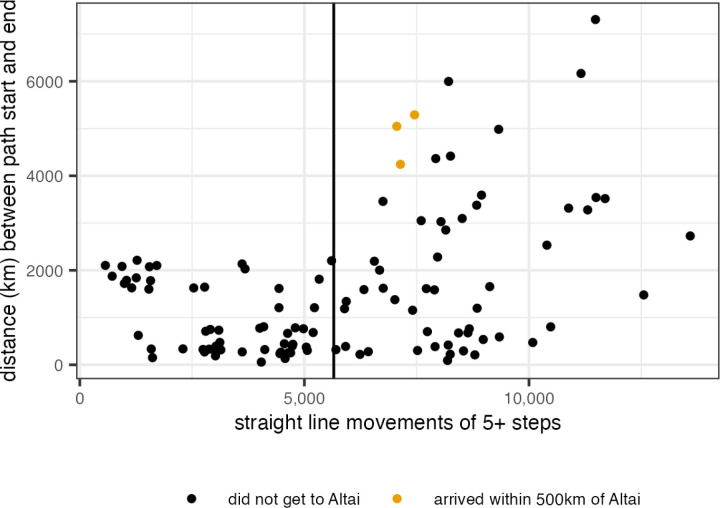
Counts of straight-line movements, i.e. segments of the movement path with no angle change, that are five steps or longer by net distance traveled in kilometers. Successful model runs that resulted in movement paths reaching within 500 km of the Altai site cluster are shown in orange. The median number of straight line movements is shown with the vertical black line.

As suggested by previous research on dispersal [41], our successful routes of rapid dispersal involve many long-distance linear movements (Fig 4). These long-distance movements are typically located along linear conduits like rivers and river valleys. One of the most typically traveled pathways is via the Volga-Kama corridor to reach the Urals, crossing the mountains along the Chusovaya and then following the Ob and Irtysh southeast toward the Altai.

### Effect of starting point

Because sites on both sides of the Caucasus are thought to have been part of a larger glacial refugium, we tested dispersal patterns that started from both the north and the south sides of the mountain range. In our model, no successful runs used a southern route through the Caspian corridor [42], and several of the routes starting south of the Caucasus turned north. One of the successful runs during MIS 5e started from the southern side of the Caucasus Mountains, but only reached the Altai site cluster after turning north and following the arc described above.

### Effect of paleogeography

Many of the previous calculations of least cost paths have ignored the major changes in landscape that followed the climatic changes associated with the end of the Last Interglacial. In particular, the re-routing of the hydrological network from the Kara Sea to the Aral Sea, currently estimated during MIS 5a [43] could have affected landscape connectivity. We therefore tested the effect of lake damming and water level rise in the hydrological network to reconstruct possible paleorivers and paleolakes in a systematic way. Given that following river valleys appears to be a characteristic of successful runs, paleolake flooding would be expected to create additional pathways for movement along new river conduits [24]. Contrary to this expectation, our study shows that these simulated water bodies acted as barriers rather than conduits for dispersal eastward. For example, Caspian Sea transgressions prevent movement out of the Caucasus Mountains during cooler periods. This may be a limitation of our method for reconstructing the paleohydrological network. Future work should be done to refine these reconstructions for fully understanding their impact on dispersal.

### Estimated arrival times

We estimate how long it takes the agents to reach the Altai by counting the number of movements they make. In each of the successful dispersals, it takes between 925,000 to 1 million steps before arriving by the Altai site cluster (Table 1). This translates to approximately 1 million kilometers of movement. Using ethnographic data [39] about how far hunter-gatherer populations move within a year (S9 Table in S1 Appendix), we estimate that Neanderthals could have arrived in the Altai within 2000 years of initial movement out of the Caucasus Mountains. Estimates of Neanderthal energetics suggest that Neanderthals would have moved less per year compared to modern humans [44], so it is possible that Neanderthal dispersals would have taken slightly longer than 2000 years. However, the model still suggests dispersal could have occurred relatively quickly. Such a dispersal may have left few vestiges along the route, as Neanderthals would have spent relatively little time in any one location.

**Table 1:** Number of agent steps and estimated number of years taken for movement paths to arrive within 500 kilometers of Altai site cluster.

Path start location	Marine Isotope Stage	Number of steps to reach Altai sites	Estimated number of years to reach Altai sites
Northern Caucasus	MIS 5e	923,439	1594.886
Southern Caucasus	MIS 5e	1,103,612	1906.066
Southern Caucasus	MIS 3	1,105,725	1587.545

### Areas of redundant movement

Across the model runs that make it out of the Caucasus Mountain region, agents frequently traverse river valleys in the Eastern European Plain like those of the Volga and Kama (see S10 Fig in S1 Appendix). Agents also frequently move in the Irtysh River valley near the northern Kazakhstan border; this is the area that typically funnels agents into the Altai region. There is frequent movement in the Turgai Valley from the northern Kazakhstan border into the Turgan Lowlands, corresponding with the location of Middle Paleolithic surface deposits in Kazakhstan [45–47]. Agents also frequently move around the Caspian Sea basin during regression periods, so these sites would be underwater today.

## Discussion and conclusions

Our results suggest that Neanderthals could have quickly dispersed from the Caucasus Mountains to the Altai Mountains via a northern route during MIS 3 and MIS 5e. Consistent eastward movement out of Western Eurasia occurs across most modeled scenarios even though agents have no goal destination and can freely pick a direction of movement. This suggests that Neanderthal dispersal to the Altai is an inevitable outcome of local movement decisions defined by geography.

The results of our analysis correspond with archaeological evidence from Chagryskaya and Okladnikov Caves that demonstrates that the Altai was occupied by Neanderthals by at least 59,000 years ago, firmly within MIS 3 [6,10]. Genetic evidence also suggests a rapid expansion of Neanderthals from Europe around 100–115 ka [3,10], which is at the end of the Last Interglacial or MIS 5e. The close correspondence of our modeled routes with the archaeological and genetic record highlights the unique strength of using agent-based least-cost path modeling to explore Neanderthal long-distance movements.

Most modeled routes that disperse out of Western Eurasia do so by moving north through the Ural Mountains. Current archaeological evidence limits occupation there to the later stages of MIS 3 [48–51], yet our model suggests Neanderthals would have been able to move into this region during the warm MIS 5 substages and even during MIS 4. Future fieldwork there may reveal older occupations.

While previous research suggested that dispersal might have been facilitated by changes in hydrology during the colder periods, our study shows that dispersal likely occurred during the warmer periods, even when accounting for these geographic changes. Dispersal during warmer periods matches expectations based on reconstructions of Neanderthal niche space that show an increase in habitat suitability in the northern parts of Eastern Europe and Central Asia during warmer marine isotope stages [52].

Neanderthals taking a northern route into Central Asia may have been expanding into Denisovan territories. Recent studies reconstructing the distribution of Denisovans suggest this species could have occupied much of the Eurasian Steppe, particularly between the Urals and the Altai, during MIS 5e and MIS 3 [53]. The timing and locations of our modeled routes would therefore allow for inter-species encounters, which is consistent with the genetic evidence for multiple interbreeding events between Neanderthals and Denisovans [54]. The potential for overlapping ranges also suggests that some of the Middle Paleolithic sites we know from southern Siberia may be Denisovan sites or even sites occupied by mixed groups, as suggested by the first-generation Denisova 11 hybrid individual [54], dated to approximately 90 ka. The archaeology of this region will likely provide important insights into the genetic and cultural consequences of these inter-species interactions.

Another area of interest is the Turgai Valley of Kazakhstan to the north of the Aral Sea. This region is visited four times during simulations under MIS 5e, MIS 5c, and MIS 4 conditions. Here, a large number of Mousterian occupations are documented in surface assemblages [45–47] despite a lack of stratified sites [55]. Future research in this region could provide important data on Neanderthal dispersals.

Despite good agreement between our model and archaeological data, a final test of the model requires more sites with a clear Neanderthal association. Some of our modeled routes bring agents close to Obi Rakhmat, Teshik-Tash, and Anghilak – the sole sites in Central Asia with Neanderthal remains [1,56,57]. Agents always enter the southern parts of Central Asia from the north, contradicting previous modeling that suggests Neanderthals reached Obi Rakhmat and Teshik-Tash by dispersing along the Southern Caspian Corridor [42]. Our model offers no southern route. It is possible that research will prove the existence of a southern dispersal route for Neanderthals. Archaeological research continues to identify sites with Middle Paleolithic assemblages in Iran [e.g., 58–68], including some with hominin fossils [69–71]. As we learn more about the Middle Paleolithic of this region and adjacent areas, like Turkmenistan and Afghanistan, we may learn more about the Neanderthal range as it expanded during the Late Pleistocene, although southeastern range expansions may be unrelated to northeastern ones. Future iterations of our model could seed Neanderthal populations out of locations further south than the Caucasus, such as the Iranian Plateau or Iraq, to test if a different origin point would result in southern routes across Eurasia.

It is important to note that this model is a simplification of a real-world process. For example, our model does not account for every external factor that may have affected how Neanderthals were able to move within this landscape. Factors such as access to resources [e.g., 72], distance to water sources [e.g., 65,73,74], annual or seasonal weather patterns and climate change [e.g., 75–77], vegetation preferences [e.g., 78], location of previous occupations [79], and others [41] may have played important roles in dictating daily movement decisions of dispersing Neanderthals. Furthermore, our cost surfaces are static representations of dynamic landscapes. This makes it impossible to include short-term or sudden changes to the landscape [e.g., 80,81] or for the agents to adapt their movement strategies depending on where they are [16]. Future use of this model will incorporate these diverse factors for developing new cost rasters; this will allow us to identify new possible dispersal routes and test hypotheses about what factors structure movement.

The results of the agent-based least cost path model demonstrate that rapid dispersal of Neanderthals from Eastern Europe to the Altai via a northern route would have been possible during multiple time periods after the retreat of the MIS 6 glaciers. Moreover, traversing the Eurasian steppe was facilitated by river valleys that would have directed random daily foraging movements, leading to eastward expansion. These results demonstrate a remarkable east-west geographic connectivity of northern Eurasia via river corridors. This highlights the strength of this methodological approach to generate specific archaeologically-testable hypotheses for pathways humans would have taken within vast landscapes. Where ecological niche and species distribution modeling allow us to define large areas suitable for habitation, an AB-LCP approach mimics ethnographic expectations of wayfinding in unknown landscapes [24], leading to the discovery of possible movement paths that made successful long distance treks into new areas possible.

## Supporting information

S1 Appendix Supporting information and figures. This appendix includes the ODD for the agent-based model, data tables for the archaeological sites referenced, data tables for the lake levels used for paleohydrological reconstructions, further details on the methods used, and additional figures to support the results.
(DOCX)
