# Supplementary material for: Agent-based simulations reveal the possibility of multiple rapid northern routes for the second Neanderthal dispersal from Western to Eastern Eurasia
Source: PLoS One. 2025 Jun 9;20(6):e0325693. doi: 10.1371/journal.pone.0325693 (PMC12148083; doi:10.1371/journal.pone.0325693)
Supplement: S1 Appendix — This appendix includes the ODD for the agent-based model, data tables for the archaeological sites referenced, data tables for the lake levels used for paleohydrological reconstructions, further details on the methods used, and additional figures to support the results. (DOCX) [file pone.0325693.s001.docx]

S1 Appendix

**Supplemental Information for: Agent-based dispersal simulations reveal the possibility of multiple rapid northern routes for the second Neanderthal dispersal from Western to Eastern Eurasia**

Emily Coco & Radu Iovita

## Overview, Design Concepts, and Details for ab-lcp-Levy_v1_hpc

The following description of the recycling agent-based model follows the ODD protocol for describing individual- and agent-based models [1–3].

**Purpose**

The main purpose of this model is to simulate possible movement across Central Asia during different climatic conditions during the Pleistocene.

**Entities, state variables, and scales**

This model consists of two primary entities: mobile hiker agents and patch agents.

Each patch agent is given a cost value, which determines how difficult it is to access that patch. Some patches are “impassable” and cannot be occupied by hiker agents. These cost values are derived from an imported ASCII cost raster that was created in QGIS. At the beginning of model run, one patch is designated the start patch and one patch is designated as the end patch.

During each model run, there is only one hiker agent. Hiker agents begin at the start patch and then make movements to adjacent patch agents based on the cost values of those patches. The number of movements a hiker agent makes is determined by a step length for each tick of model run.

The global state variables are in S1 Table. The hiker state variables are in S2 Table. The patch state variables are in S3 Table.

**S1 Table. Global state variables.** * = User-defined

| *Variable name* | *Description* |
| --- | --- |
| map-resolution-km* | The resolution of the DEM (km) |
| patch-size-km* | The resolution at which the user wants to run the model (km) |
| view-radius-km* | Determines how far an agent’s field of vision is for establishing what parts of the landscape are known |
| levy-mu* | The mu parameter for determining step length |
| explore?* | Determine whether agents move further when in unexplored territory |
| time-period* | Determines cost raster used based on Marine Isotope Stage |
| limit-ticks* | Sets a limit on how many ticks the model can run for |
| basemap | Takes on the imported cost values using the GIS extension of NetLogo |
| start-area | Defines the patches where the hiker can start based on which patches overlap with the provided shapefile |
| origin | Identifies patch ID of where the hiker sprouts |
| coord-start | Identifies x-coordinate and y-coordinate of where the hiker sprouts |
| min-cost | Identifies the minimum cost value of the landscape |
| max-cost | Identifies the maximum cost value of the landscape |
| res-m | Calculates the patch resolution in meters |
| hiker-n | Identifies the hiker ID of the current hiker in the model |
| hiker-status | Tracks whether the hiker is alive |
| view-radius | Resized field of vision parameter based on basemap resolution |
| coord-list | List of coordinates for each grid square a hiker occupies during model run |
| num-steps | Counts number of steps an agent takes |
| file-1 | Name of file for exporting path from plot (only local model version) |
| file-2 | Name of file for exporting path from hiker’s *coord-list* |
| stamp1 | Random float number to make distinct output CSVs |

**S2 Table. Hiker state variables.**

| *Variable name* | *Description* |
| --- | --- |
| patch-vision | Patches the hiker considers when picking a patch to move to |
| winner-patch | Patch the hiker selects to move to |
| step-lengths | List of length of steps hiker takes during model run |
| cur-step-length | Length of current step the hiker is taken based on the Levy walk equation |

**S3 Table. Patch state variables.**

| *Variable name* | *Description* |
| --- | --- |
| cost | Cost value import as ASCII from the cost raster |
| impassable | Determines if patch can be occupied by a hiker or not |
| patch-counter | Records how many ticks since patch was occupied |
| known? | Determines if a patch has previously been in an agent’s field of vision |

**Process overview and scheduling**

At each tick of the model, the model first checks to see if the hiker has died or if the tick limit has been reached. If neither of those conditions are true, the time step proceeds.

During each tick, the hiker performs a find least cost path procedure. First, the hiker finds an adjacent square in its field of vision that has the lowest cost and faces it. The hiker then determines how many steps it will take in that direction using the Lévy walk function and a *mu* parameter, following the methodology used by Davies and colleagues [4]. The step length is determined by the following equation:

$$P\left( l \right)=l^{-\mu}$$

where *P(l)* is the probability of step length, *l*. Using this equation allows us to vary how long agents spend inside the window of observation by varying the value of μ (mu). When mu is greater than or equal to 3, the likelihood of step lengths greater than 1 becomes increasingly small, leading to the agent spending more time inside the modeled landscape. As mu approaches a value of 1, there is a higher probability of longer step lengths, meaning the agent is more likely to exit the landscape quickly. For each step in the step length, the hiker performs the same procedure: find an adjacent square with the lowest cost and move to it. Once the step length has been completed, the model ticks and the movement process begins again.

**Design concepts**

*Basic principles*

This model tries to represent human movement more accurately in a topographically complex environment. This model builds on the idea of basic least cost path analysis that requires knowledge of the whole landscape to find a path between two points.

*Emergence*

The path itself emerges from a hiker’s movement decisions at each tick and step.

*Objectives*

The hiker will always move to patches with the lowest cost among the patches directly adjacent to the hiker.

*Learning*

Patches update their *patch-counter* once they have been occupied. Hikers change their temporary target (*winner-patch*) once they move to it.

*Sensing*

Hikers know the cost of their adjacent patches, which allows them to choose patches with the lowest costs.

*Stochasticity*

Hiker step length is determined randomly via Lévy walks. The direction that hikers face at the beginning of their Lévy walk is also randomly determined from all the patches adjacent to the hiker.

*Observation*

The plot keeps track of the x and y coordinates of a hiker’s location with each step the hiker takes.

**Initialization**

The world is a grid of patches that does not wrap around. The cost of each patch is determined by an ASCII cost raster. Which raster is loaded is determined by the *time-period* parameter. Before setup, the user tells the model the resolution of the map provided (in kilometers) with the *map-resolution-km* parameter. The user also decides the resolution of the landscape by setting the *patch-size-km* parameter. When these two variables differ, the cost of patches is determined via the bicubic_2 method. Using the difference between map resolution and patch size resolution, the *view-radius-km* parameter is also rescaled. Once the landscape has been initialized, a single hiker is placed on the landscape at a random patch within a given start area that is imported into the model as a shapefile. Parameters used during model runs can be found in S4 Table.

**S4 Table. Parameter settings used for paper.**

| **Parameter** | **Setting** |
| --- | --- |
| limit-ticks | 400,000 |
| output? | TRUE |
| lost-output? | TRUE |
| map-resolution-km | 1 |
| patch-size-km | 1 |
| view-radius-km | 5 |
| levy-mu | 1 |
| explore? | TRUE |
| time-period | MIS 6 (high & low water), MIS 5e, MIS 5d (high & low water), MIS 5c, MIS 5b (high & low water), MIS 5a, MIS 4 (high & low water), MIS 3 |

**Input data**

Cost rasters and start/end areas are imported into the model in ASCII and shapefile formats, respectively. Both must have the same projection.

**Submodels**

To stp-hiker:

Creates the hiker, represented as a violet person figure. The hiker’s pen is down to follow their path.

The *hiker-n*, *origin*, and *coord-start* variables are set. An empty list of *step-lengths* is also created.

To go:

Determines whether or not to end the model run based on whether the hiker died in the previous tick or the tick limit has been reached.

Reduces the *patch-counter* variable of each patch by 1 to eventually allow agents to walk on them again.

Calls the find-least-cost-path function.

Exports an in-progress version of the list of coordinates an agent has occupied.

Advances the ticks.

To find-winner-patch [#cone-radius]:

Hiker finds a *winner-patch* to face from the patches in a radius determined by the *#cone-radius* parameter. The chosen patch must be walkable (i.e. not *impassable*) and have a *patch-counter* value of 0.

The hiker also checks to see if any of the possible patches to move to are unknown (i.e. *known?* is false) patches, meaning those patches that have never been the hiker’s view radius before. If there are unknown patches, *winner-patch* is set to the patch with lowest cost from those unknown patches. If not, *winner-patch* is set to the patch with lowest cost that is walkable and has a *patch-counter* value of 0.

To find-least-cost-path:

Hiker calls the *find-winner-patch* function with a *#cone-radius* of 360-degrees.

The hiker then faces the *winner-patch* and calls the *get-step-length* function. Then the hiker moves via the *move* function.

To get-step-length:

Calculates the *cur-step-length* via the Lévy walk function (see above).

If the *explore?* parameter is on, the hiker assesses whether more than half of its surrounding squares have a *patch-counter* of 0. If this is true, the hiker doubles its *cur-step-length*. The hiker also rounds *cur-step-length* up to the nearest whole number.

Adds the new *cur-step-length* to the list of *step-lengths* tracked throughout the model.

To move:

For each step in *cur-step-length*: Moves hiker to the *winner-patch* and updates the plot. Sets the *known?* parameters of patches within the *view-radius* to true. Also sets the patch counter of the *winner-patch* to 100 to designate it has already been walked. Finds a new *winner-patch* via the *find-winner-patch* function.

If a hiker’s path takes it to the edge of the world, the model stops.

After all the steps have been completed, hiker sets all immediately adjacent patches to unknown (i.e. *known?* is false) to allow for more freely picking a new heading on the next tick based on the *find-winner-patch* function.

To update-colors:

Patches are given a color based on their cost.

For land patches, a green color scale is determined by the maximum and minimum cost values of the imported raster. For impassable patches, the color is blue.

To export-path:

Export the x and y coordinates of the hiker (the plot) to a CSV file in an output folder.

To export-coord-list:

Export the list of x and y coordinates of the hiker from a running list of coordinates that is updated each time the hiker moves to a new square. This list is exported to a CSV file in an output folder using the CSV extension of NetLogo.

## Middle Paleolithic sites

**S5 Table. Middle Paleolithic and/or Neanderthal archaeological sites dated between MIS 6 and MIS 3.**

| **Site ID** | **Country** | **Latitude** | **Longitude** | **Period** | **Reference** |
| --- | --- | --- | --- | --- | --- |
| Gazma | Azerbaijan | 39.512778 | 45.174444 | MIS 3 | Zeynalov et al., 2023 [5] |
| Azokh 1 | Azerbaijan | 39.62 | 46.99 | MIS 5 | Fernández-Jalvo et al., 2016 [6,7] |
| Bronze Cave | Georgia | 42.27 | 42.85 | MIS 3 | Adler et al., 2008; Pinhasi et al., 2012 [8,9] |
| Sakaija | Georgia | 42.28 | 42.757 | MIS 3 | Moncel et al., 2015; Pinhasi et al., 2012 [9,10] |
| Ortvala | Georgia | 42.284 | 42.762 | MIS 3 | Pinhasi et al., 2012 [9] |
| Bondi Cave | Georgia | 42.33 | 43.28 | MIS 3 | Pleurdeau et al., 2016 [11] |
| Djruchula Cave | Georgia | 42.34 | 43.34 | MIS 5-6 | Adler and Tushabramishvili 2004; Mercier et al., 2010 [12,13] |
| Bawa Yawan | Iran | 34.64 | 46.93 | MIS 3 | Heydari-Guran et al., 2021 [14] |
| Mirak | Iran | 35.469 | 53.431 | MIS 3 | Vahdati Nasab et al., 2019 [15] |
| Ghār-e Boof | Iran | 30.2839 | 51.4352 | MIS 3-5 | Ghasidian et al., 2017; Heydari et al., 2021 [16,17] |
| Wezmeh Cave | Iran | 34.056 | 46.645 | MIS 3? | Trinkaus et al., 2008; Zanolli et al., 2019 [18,19] |
| Kaldar Cave | Iran | 33.557 | 48.293 | MIS 4 | Bazgir et al., 2022 [20] |
| Qaleh Kurd Cave | Iran | 35.797222 | 48.857222 | MIS 6? | Vahdati Nasab et al., 2024 [21] |
| Shanidar | Iraq | 36.831593 | 44.221083 | MIS 3-4 | Trinkaus 1983; Pomeroy et al., 2020 [22,23] |
| Amud | Israel | 32.87222 | 35.501667 | MIS 4 | Hovers 1998; Valladas et al., 1999 [24,25] |
| Kebara | Israel | 32.558278 | 34.937306 | MIS 3-4 | Schwarcz et al., 1989; Rebollo et al., 2011 [26,27] |
| Tabun | Israel | 32.6705 | 34.9655 | MIS 5 | Grün and Stringer 2000; Coppa et al., 2005 [28,29] |
| Sel’ungur | Kyrgyzstan | 39.95333 | 71.32528 | MIS 5 | Krivoshapkin et al., 2020 [30] |
| Bisnik Cave | Poland | 50.426351 | 19.664764 | MIS 5 | Cyrek et al., 2014 [31] |
| La Adam Cave | Romania | 44.464437 | 28.471511 | MIS 3-5 | Dobrescu et al., 2008; Iovita et al., 2014 [32] |
| Sukhaya Mechetka | Russia | 48.830713 | 44.648973 | MIS 3 | Praslov and Kuznetsova, 2020 [33] |
| Mezmaiskaya | Russia | 44.166 | 40 | MIS 3 | Golovanova et al., 1999 [34] |
| Strashnaya | Russia | 51.17 | 83.03 | MIS 3 | Derevianko, 2015 [35] |
| Ust-Karakol | Russia | 51.3824 | 84.6907 | MIS 3 | Derevianko et al., 2003 [36] |
| Ust-Kanskaya | Russia | 50.912 | 84.8129 | MIS 3 | Lesage et al., 2020 [37] |
| Okladnikov | Russia | 51.73333 | 84.03333 | MIS 3 | Derevianko and Markin 1992 [38] |
| Barakaevskaya | Russia | 44.28001 | 40.52667 | MIS 3 | Faerman et al., 1994; Golovanova and Doronichev, 2003 [39,40] |
| Nosovo I | Russia | 47.283406 | 38.68247 | MIS 3 | Praslov, 1968 [41] |
| Garchi I | Russia | 59.03361 | 56.16694 | MIS 3 | Pavlov, 2008; Svendsen et al., 2010 [42,43] |
| Denisova | Russia | 51.3976 | 84.6775 | MIS 3-7 | Jacobs et al., 2019 [44] |
| Imanai | Russia | 53.03333 | 56.51667 | MIS 3? | Kotov et al., 2020 [45] |
| Byzovaya | Russia | 65.02361 | 57.42 | MIS 3? | Slimak et al., 2011 [46] |
| Treugol'naya | Russia | 43.9 | 41.2 | MIS 4 | Blackwell et al., 2005 [47] |
| Chagyrskaya | Russia | 51.422 | 83.1263 | MIS 4 | Derevianko et al., 2013 [48] |
| Kara-Bom | Russia | 50.7228 | 85.5743 | MIS 4 | Slavinsky et al., 2016 [49] |
| Shlyakh | Russia | 49.59833 | 43.70139 | MIS 5 | Hoffecker et al., 2019 [50] |
| Khotylevo | Russia | 53.3421437 | 34.1194444 | MIS 5 | Otcherednoi et al., 2018 [51] |
| Rozhok I | Russia | 47.14787 | 38.41469 | MIS 5 | Hoffecker et al., 2020 [52] |
| Il'skaya | Russia | 44.81406 | 38.55291 | MIS 5 | Shchelinskiĭ, 2012 [53] |
| Saradj-Chuko | Russia | 43.63122 | 43.30046 | MIS 5 | Doronicheva et al., 2020 [54] |
| Hadjoh-2 | Russia | 44.31303 | 40.24172 | MIS 5 | Doronicheva et al., 2018 [55] |
| Bogdanovka | Russia | 52.40028 | 59.06694 | MIS 5? | Shirokov et al., 2011 [56] |
| Matuzka | Russia | 44.19318 | 39.89646 | MIS 5 | Golovanova and Doronichev, 2003 [40] |
| Khudji | Tajikistan | 38.618 | 68.21 | MIS 3 | Trinkaus et al., 2000; Fagernäs et al., 2024 [57,58] |
| Zaskalnaya VI | Ukraine | 45.16 | 34.66 | MIS 3 | Stepanchuk et al., 2017 [59] |
| Antonovka I | Ukraine | 47.88222 | 37.35778 | MIS 3-4 | Stepanchuk and Sapozhnikov 2010 [60] |
| Antonovka II | Ukraine | 47.88222 | 37.35778 | MIS 3-4 | Stepanchuk and Sapozhnikov 2010 [60] |
| Kiik-Koba | Ukraine | 45.05 | 34.3 | MIS 3? | Trinkaus et al., 2008; Majkić et al., 2018 [61,62] |
| Kurdyumovka | Ukraine | 48.46833 | 37.96 | MIS 4 | Stepanchuk and Sapozhnikov 2010 [60] |
| Korneev Yar | Ukraine | 48.919937 | 38.073658 | MIS 5 | Stepanchuk and Sapozhnikov 2010 [60] |
| Anghilak | Uzbekistan | 39.285 | 66.687 | MIS 3 | Glantz et al., 2008 [63] |
| Teshik-Tash | Uzbekistan | 38.29 | 67.05 | MIS 3? | Krause et al., 2007; Nishiaki and Aripdjanov 2021 [64,65] |
| Obi Rakhmat | Uzbekistan | 41.56911 | 70.13342 | MIS 5 | Krivoshapkin et al., 2010 [66] |
| Kulbulak | Uzbekistan | 41.00861 | 70.00611 | MIS 5 | Kolobova et al., 2012; Pavlenok et al., 2018 [67,68] |

## Methods

### Building cost surfaces

The cost rasters for this study were built using data from digital elevation models (DEMs), paleoclimate data on annual precipitation, Pleistocene glacier extents, extents of modern rivers, lakes, and oceans, and paleogeographic data on Pleistocene glacier lake outbursts. The sources for this data can be found below in S6 Table. All the derived data used to construct the cost raster is available at <http://doi.org/10.17605/OSF.IO/ZF9MP>.

**S6 Table. Data sources for developing cost rasters.** Raster resolution provided for raster datasets.

| **Dataset** | **Resolution** | **Reference** |
| --- | --- | --- |
| DEM | 15 arc seconds | OpenTopography STRM15+ [69] |
| Annual precipitation | 0.5 degrees | pastclim R package [70] |
| Glacier extents |  | Batchelor et al 2019 [71] |
| Modern rivers and lakes |  | HydroSheds [72,73] |
| Modern oceans |  | Marine Regions [74] |

To begin building cost surfaces, we first resampled the raw DEM using the Warp tool in QGIS to reproject the DEM into a Pseudo-Mercator projection (EPSG:3857) and set the output raster at a resolution of 1000 m (1 km). All other datasets were converted into rasters as needed and resampled to match the resampled DEM during raster calculator operations (see cost raster creation description file at <http://doi.org/10.17605/OSF.IO/ZF9MP>).

We used slope-dependent cost functions to estimate energy expenditure by a walking individual , which is a more appropriate model than Tobler’s hiker function or backpacker functions [75,76]). We used Llobera and Sluckin’s (LS) walker cost function, which is based on mathematical slope, that is percent slope divided by 100 [75,77]. The slope-based costs were used as a base map to which other costs were added.

In order to model the potential difficulty of crossing deserts, we increased the base cost of areas that received less than 250mm of annual precipitation, following Li et al’s strategy [78] to allow short movements across these regions [79,80]. In Li et al’s least cost path analysis, they gave arid areas a cost equivalent to a slope of 15 degrees. For our study, we decided to instead increase the cost for the cells by the amount equivalent to an increase from a slope of 0 degrees to a slope of 15 degrees [81–83], which for the LS cost function is equivalent to a 74% increase in cost.

Large bodies of water and glaciers are considered barriers in our model. We used the best-estimate glacier extent data from Batchelor et al [71] to create glacial barriers for MIS 6, all MIS 5 substages, MIS 4, and MIS 3. Glaciated areas were given a negative infinity value (-99999) in our cost raster, which the model identifies and labels as impassable. Similarly, we used modern lake and ocean data to produce some of our water barriers (see description of further water barriers below). These water bodies were also given an impassable value. For rivers, we used average long-term water discard estimates from the HydroRIVERS dataset to approximate river width based on Moody and Troutman’s power law relating river width and discharge [84,85]. River center lines were buffered to these widths. Rivers with a width greater than a kilometer were given an impassable value. Rivers with width less than a kilometer were further buffered by 50m and the total area was given a cost consistent with an increase from 0 to 15 degrees of slope (approx. 74% increase in LS cost) to allow for some river crossings to take place following previously used methodologies [81–83].

There are many studies that demonstrate dramatic paleogeographic changes in Central Asia during the Pleistocene [86–92]. We surveyed these studies and others to simulate paleolakes and paleochannels using the Lake Flood function in QGIS [93]. These lake levels were derived from a literature review of water body levels from the early Holocene to MIS 6 (S7 Table). The lake levels used to create the flooded lakes can be found in S8 Table. In some MIS periods, there are discrepancies in the estimated lake levels. In these cases, we developed cost rasters based on high lake level estimates and low lake level estimates. For MIS periods where some lake levels are not currently estimated, we used lake levels from the nearest comparable time period (i.e., nearest glacial or interglacial).

**S7 Table. Estimated water body levels from early Holocene to MIS 6.** Rows in italics were not used for the analyses in this paper.

| Water Body | Level  (m asl) | Dates | Period | MIS Stage | Citation(s) |
| --- | --- | --- | --- | --- | --- |
| Aral Sea | 57 |  | early Holocene |  | Svitoch, 2009 [94] |
| *Kara Sea* | *60* |  | *Late Weichselian* | *MIS 2* | Panin et al., 2020 [91] |
| *Volga River* | *140* |  |  | *MIS 2* | Panin et al., 2020 [91] |
| *Caspian* | *-20* |  | *Novocaspian transgression* | *post MIS 3* | Kurbanov et al., 2021 [95] |
| *Caspian* | *0* |  | *Late Khvalynian transgression* | *post MIS 3* | *Dolukhanov et al., 2010; Kurbanov et al., 2021* [95,96] |
| *Caspian* | *22* |  | *Middle Khvalynian transgression* | *post MIS 3* | *Dolukhanov et al., 2010; Kurbanov et al., 2021* [95,96] |
| *Caspian* | *50* | *17- 13.1 ka* | *Early Khvalynian transgression* | *post MIS 3* | *Dolukhanov et al., 2010; Kurbanov et al., 2021* [95,96] |
| Kara Sea (global sea level) | modern level |  |  | MIS 3 | Gavrilov et al., 2020 [97] |
| Black Sea | -25 |  |  | MIS 3 | Svitoch et al., 2000 [98] |
| Caspian | -140 | 27.2 ka | Atelian regression | MIS 4/3 | Dolukhanov et al., 2010; Bezrodnykh et al., 2020; Kurbanov et al., 2021 [95,96,99] |
| Barents | 40 |  |  | MIS 4 | Larsen et al., 2006 [100] |
| White Sea | 45 | 60 ka | middle Weichselian | MIS 4 | Mangerud, 2004 [87] |
| Dnieper River | 30 to 55 |  | middle Weichselian | MIS 4 | Mangerud, 2004 [87] |
| Kara Sea | 45 |  | middle Weichselian | MIS 4 | Mangerud, 2004 [87] |
| *Taimyr* | *80* | *60 ka* |  | *MIS 4* | Mangerud, 2004 [87] |
| Kara Sea (global sea level) | -30 |  |  | MIS 5a | Gavrilov et al., 2020 [97] |
| White Sea | 12 |  |  | MIS 5a | Dalton et al., 2021 [101] |
| Ob River | 90 |  | Middle Pleistocene | late MIS 5  (MIS 5b) | Panin et al., 2020 [91] |
| Aral | 70 | 70-90 ka |  | MIS 5b | Panin et al., 2020 [91] |
| Kara Sea (global sea level) | 1 |  |  | MIS 5b | Gavrilov et al., 2020 [97] |
| Manych Pass (Caspian, Black) | 26 | 90 ka | early Weichselian | MIS 5b | Mangerud et al., 2001; Mangerud, 2004 [87,102] |
| Turgai Pass (White Sea) | 126 | 90 ka | early Weichselian | MIS 5b | Mangerud et al., 2001; Mangerud, 2004 [87,102] |
| Uzboy Pass (Aral) | 57 | 90 ka | early Weichselian | MIS 5b | Mangerud et al., 2001; Mangerud, 2004 [87,102] |
| Kara Sea (global sea level) | -25 |  |  | MIS 5c | Gavrilov et al., 2020 [97] |
| Global sea level | -60 |  |  | MIS 5d | Gavrilov et al., 2020 [97] |
| Barents | 100 |  | Eemian- Boreal transgression | MIS 5d | Astakhov, 2006; Korsakova, 2009; Komatsu et al., 2016 [88,89,103] |
| Kara Sea (global sea level) | 0 |  |  | MIS 5e | Gavrilov et al., 2020 [97] |
| Caspian | -5 |  | Late Khazarian (Eemian) | MIS 5e | Költringer et al., 2021 [104] |
| Ob River | 60 |  | early Weichselian | MIS 5a-d | Astakhov, 2006 [88] |
| Yenissei River | 70 |  | early Weichselian | MIS 5a-d | Astakhov, 2006 [88] |
| *Taimyr* | *140* | *80 ka* |  | *MIS 5* | *Mangerud, 2004* [87] |
| Pechora River | 135 |  |  | MIS 6/ MIS 5c-d | Panin et al., 2020 [91] |
| Caspian | 10 |  | Late Khazar transgression | MIS 6/ MIS 5e | Yanina, 2012 [105] |
| Black Sea | 6 |  | Karangatian transgression | MIS 6/ MIS 5 | Svitoch et al., 2000 [98] |
| Kara Sea | 60 |  | Saalian- Eemian | MIS 6 | Larsen et al., 2006 [100] |
| Kara Sea | 120 |  |  | MIS 6 | Panin et al., 2020 [91] |

**S8 Table. Lake levels used for each cost raster by Marine Isotope Stage.**

| Stage | Water amount | Black Sea level (masl) | Caspian Sea level (masl) | Aral Sea level (masl) | Kara Sea level (masl) | Other levels |
| --- | --- | --- | --- | --- | --- | --- |
| MIS 3 | ~ | -25 | -140 | 57 | 0 |  |
| MIS 4 | low | -25 | -140 | 57 | 45 | Barents Sea: 40 masl, White Sea: 45 masl |
| MIS 4 | high | ~ | ~ | 57 | 45 | Barents Sea: 40 masl, White Sea: 45 masl; Dnieper River: 30 masl |
| MIS 5a | ~ | 6 | -5 | 57 | -30 | White Sea: 12 masl |
| MIS 5b | low | ~ | ~ | 70 | 1 | Ob River: 60 masl, Yenissei River: 70 masl |
| MIS 5b | high | ~ | ~ | 70 | 1 | White Sea: 126 masl; Ob River: 90 masl |
| MIS 5c | ~ | 6 | -5 | 57 | -25 |  |
| MIS 5d | low | 26 | ~ | 57 | ~ | Ob River: 60 masl, Yenissei River: 70 masl |
| MIS 5d | high | ~ | ~ | 70 | ~ | Barents Sea: 100 masl |
| MIS 5e | ~ | 6 | -5 | 57 | 0 |  |
| MIS 6 | low | 6 | 10 | 57 | 60 |  |
| MIS 6 | high | 6 | 10 | 57 | 120 |  |

### Analysis of routes

For each cost raster, we ran the model five times to understand the variation in the movement paths. We read in the list of coordinates that the agent traveled for each movement path and then counted how many times the agent visited each unique set of coordinates across each of the five model runs. This occupation count is translated into a frequency by dividing the count by the maximum number of occupations at any given set of coordinates. The frequency of occupation of each set of coordinates is exported as a raster.

We do the same procedure to identify areas of redundant movement, except that the frequency is calculated across all movement paths produced for cost raster representing MIS 3, MIS 4, MIS 5a, MIS 5c, and MIS 5e. This frequency calculation is also written as a raster. Because the highest frequencies of occupations will necessarily occur near the Caucasus Mountains because every path starts from there, we filtered out these high frequency outliers by removing coordinate pairs with occupation frequencies 1 standard deviation above the mean occupation frequency across all coordinate pairs. This removes only 3% of all occupied coordinates, primarily nearest to the Caucasus Mountains.

To identify successful movement paths, we created a 500-km buffer shapefile in QGIS around the Middle Paleolithic sites located in the Russian Altai. We then turned each path coordinate list into a shapefile that can be compared to the buffer zone. If any of the path coordinates fall within the buffer zone, the movement path is a “success.”

To estimate the arrival times of these successful runs, we identify at which step the agent first enters the buffer zone; this represents the number of steps it takes to reach the Russian Altai. To turn this number of steps into years, we calculated how many kilometers mobile populations would be expected to move on average per year from ethnographic data using Binford’s Constructing Frames of Reference dataset [106], which has been turned into an R package [107]. We calculated the first and third quartiles of estimated mean annual temperature and estimated annual precipitation across our study area for each Marine Isotope Stage using the pastclim package [70]. We used these values to query the ethnographic data to identify those fully nomadic (sed = 1) ethnographic groups whose recorded mean annual temperature (cmat) and recorded annual rainfall (crr) fall between the first and third quartiles of each Marine Isotope Stage. The results by Marine Isotope Stages are given in Supplementary Table 9.

**S9 Table. Average number of moves made per year and kilometers moved per year by ethnographic hunter-gatherer populations.**

| **Marine Isotope Stage** | **Average number of moves per year (NOMOV)** | **Average kilometers moved per year (KMOV)** |
| --- | --- | --- |
| MIS 3 | 17.29 | 696.47 km |
| MIS 4 | 16.86 | 989.57 km |
| MIS 5a | 17.43 | 698.77 km |
| MIS 5b | 18.0 | 707.96 km |
| MIS 5c | 18.3 | 704.74 km |
| MIS 5d | 17.83 | 694.55 km |
| MIS 5e | 19.0 | 579.24 km |

We also examined the frequency of straight-line movements by analyzing angle changes along the output route based on the XY coordinates of steps using the trajr package [108]. Angles of each step were calculated relative to north (angle of 0 radians) and compared to the previous angle in the step sequence to calculate angle change. The angle change values were then used to compute the length of runs along the output route with no angle change (angle change = 0). For each output route, we counted how many runs of no angle change with a run length greater than or equal to the 97.5% quantile value of the average run length. For our results, this is equivalent to any run lengths greater than or equal to 5 steps.

## Supplementary Figures


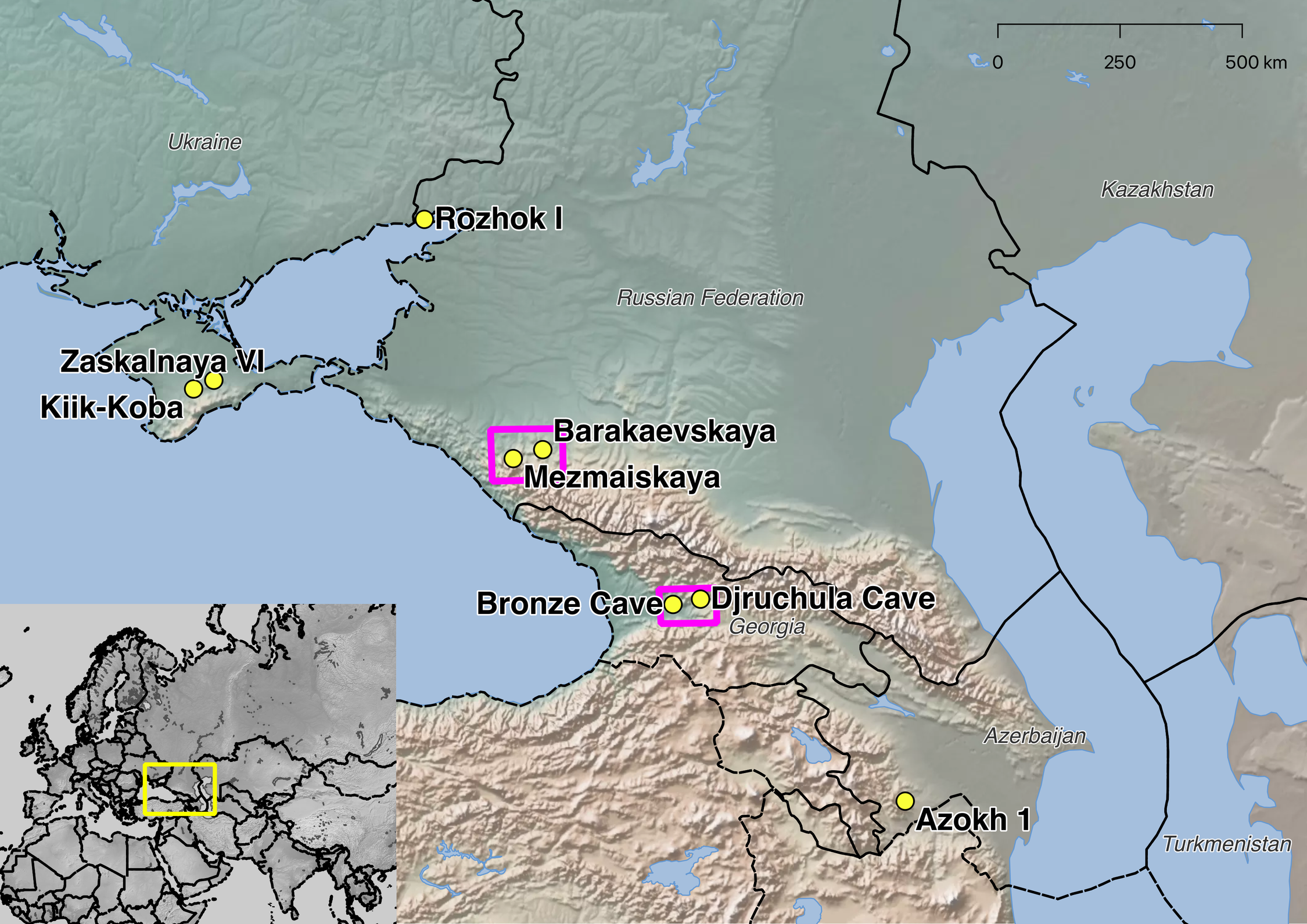


**S1 Fig.** Start areas for model runs in the Northern Caucasus (upper pink box) and Southern Caucasus (lower pink box). Base map provided by Natural Earth.


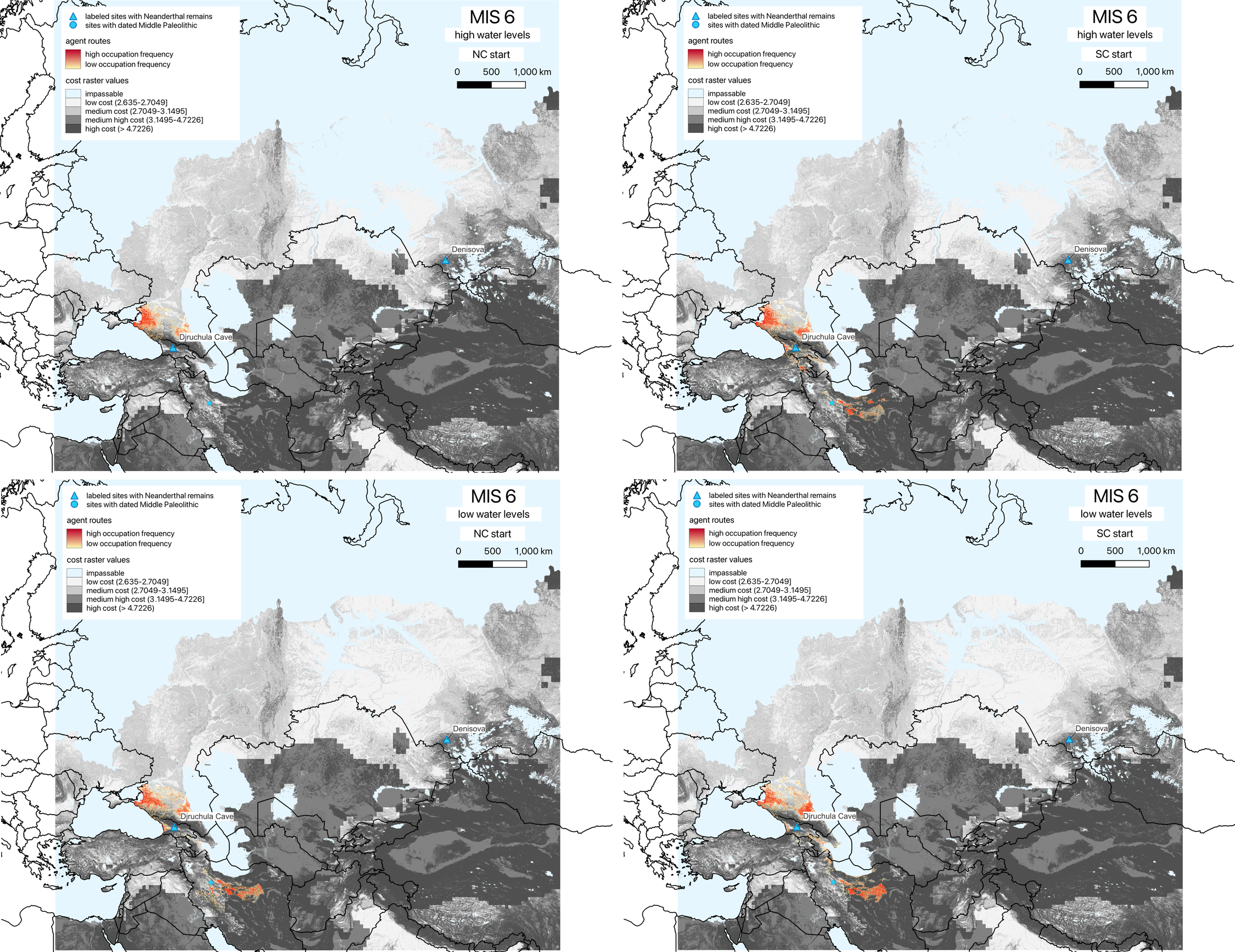


**S2 Fig.** Summary of output paths for all MIS 6 scenarios. Each image shows the frequency of agent steps within cost raster grid squares: locations visited more frequently are shown in red and those visited less frequently are shown in yellow. The base map shows the input cost raster where red is high cost and green is low cost. Cost rasters with high water levels in the left images. Cost rasters with low water levels in the right images. The top two images show output paths from a Northern Caucasus starting position. The bottom two images show output paths from a Southern Caucasus starting position.


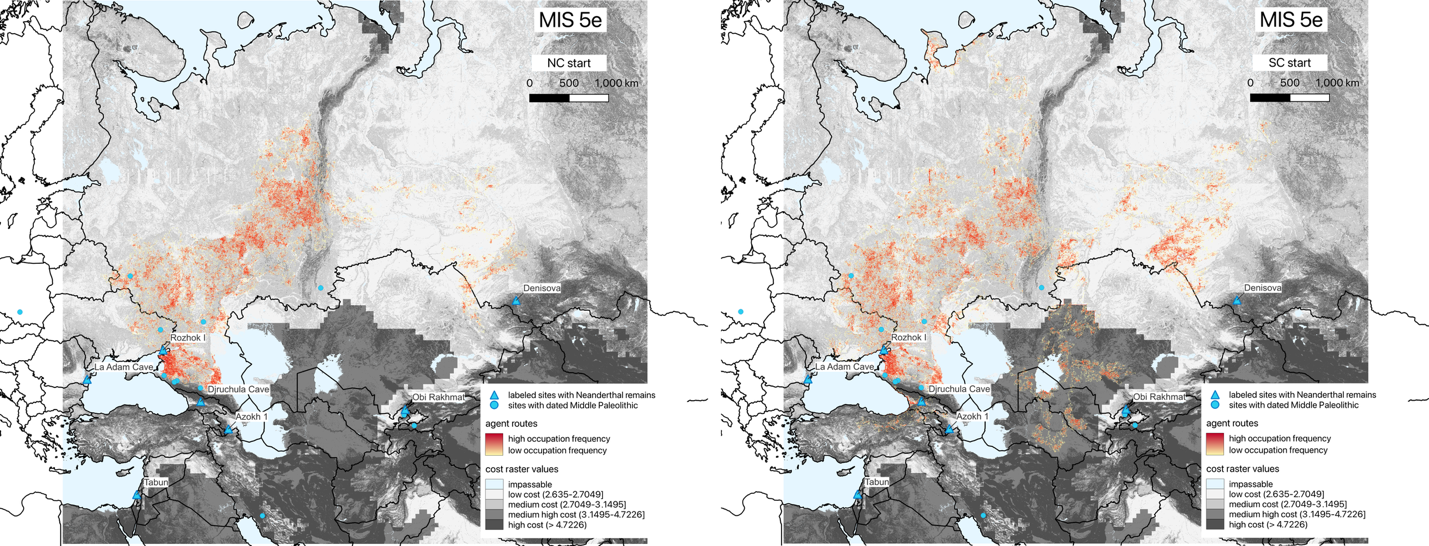


**S3 Fig.** Summary of output paths for all MIS 5e scenarios. Each image shows the frequency of agent steps within cost raster grid squares: locations visited more frequently are shown in red and those visited less frequently are shown in yellow. The base map shows the input cost raster where red is high cost and green is low cost. The left image shows output paths from a Northern Caucasus starting position. The right image shows output paths from a Southern Caucasus starting position.


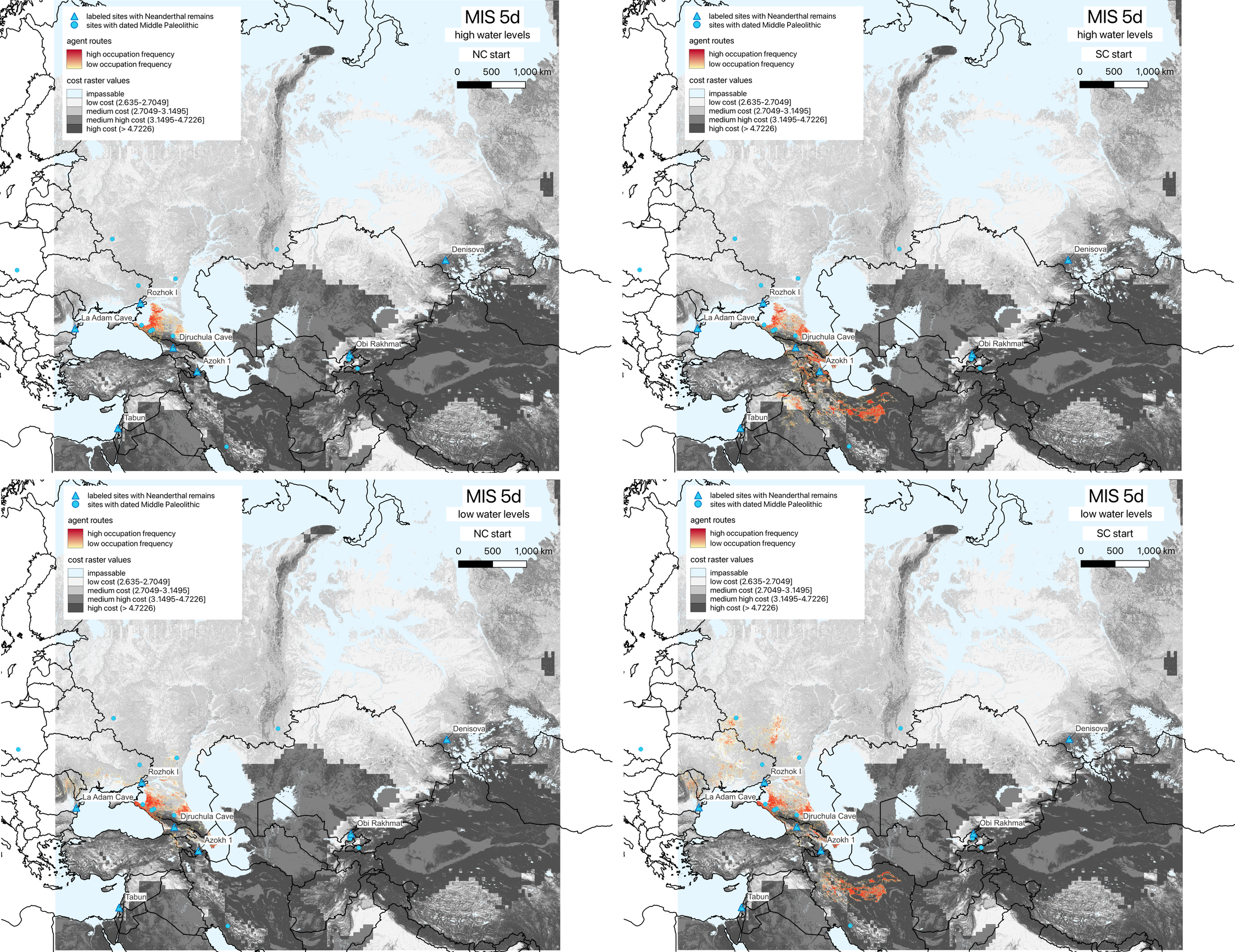


**S4 Fig.** Summary of output paths for all MIS 5d scenarios. Each image shows the frequency of agent steps within cost raster grid squares: locations visited more frequently are shown in red and those visited less frequently are shown in yellow. The base map shows the input cost raster where red is high cost and green is low cost. Cost rasters with high water levels in the left images. Cost rasters with low water levels in the right images. The top two images show output paths from a Northern Caucasus starting position. The bottom two images show output paths from a Southern Caucasus starting position.


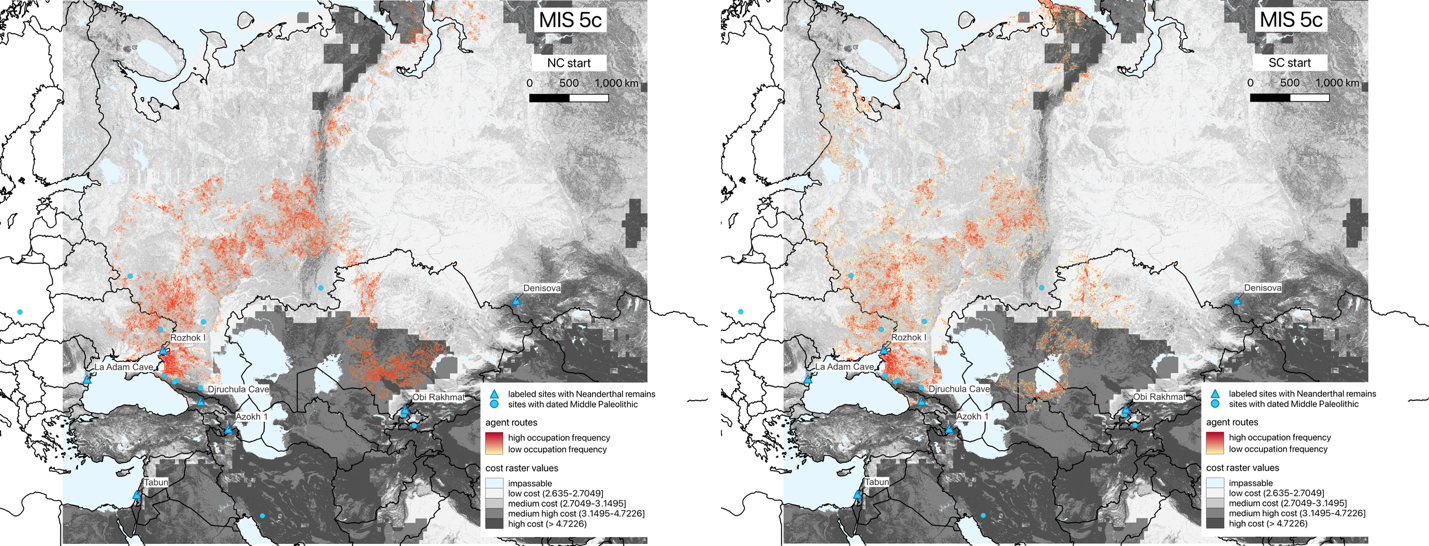


**S5 Fig.** Summary of output paths for all MIS 5c scenarios. Each image shows the frequency of agent steps within cost raster grid squares: locations visited more frequently are shown in red and those visited less frequently are shown in yellow. The base map shows the input cost raster where red is high cost and green is low cost. The left image shows output paths from a Northern Caucasus starting position. The right image shows output paths from a Southern Caucasus starting position.


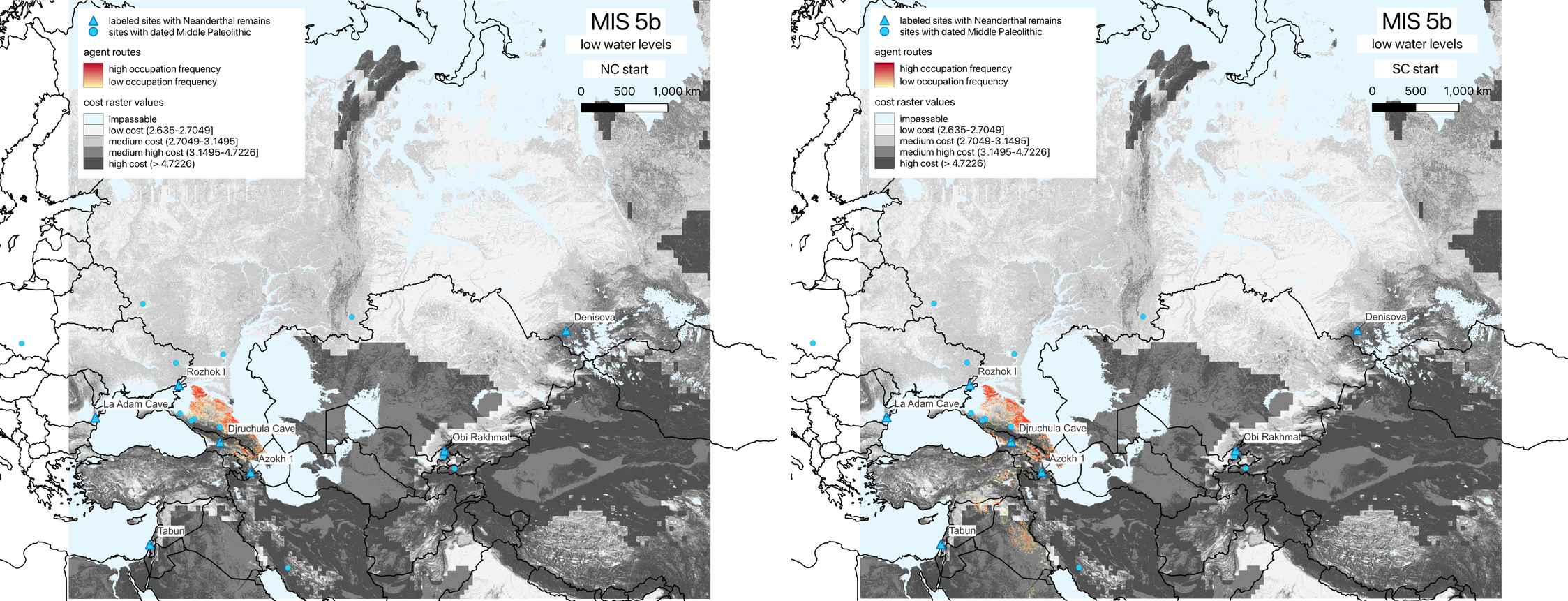


**S6 Fig.** Summary of output paths for all MIS 5b scenarios. Each image shows the frequency of agent steps within cost raster grid squares: locations visited more frequently are shown in red and those visited less frequently are shown in yellow. The base map shows the input cost raster where red is high cost and green is low cost. The left image shows output paths from a Northern Caucasus starting position. The right image shows output paths from a Southern Caucasus starting position.


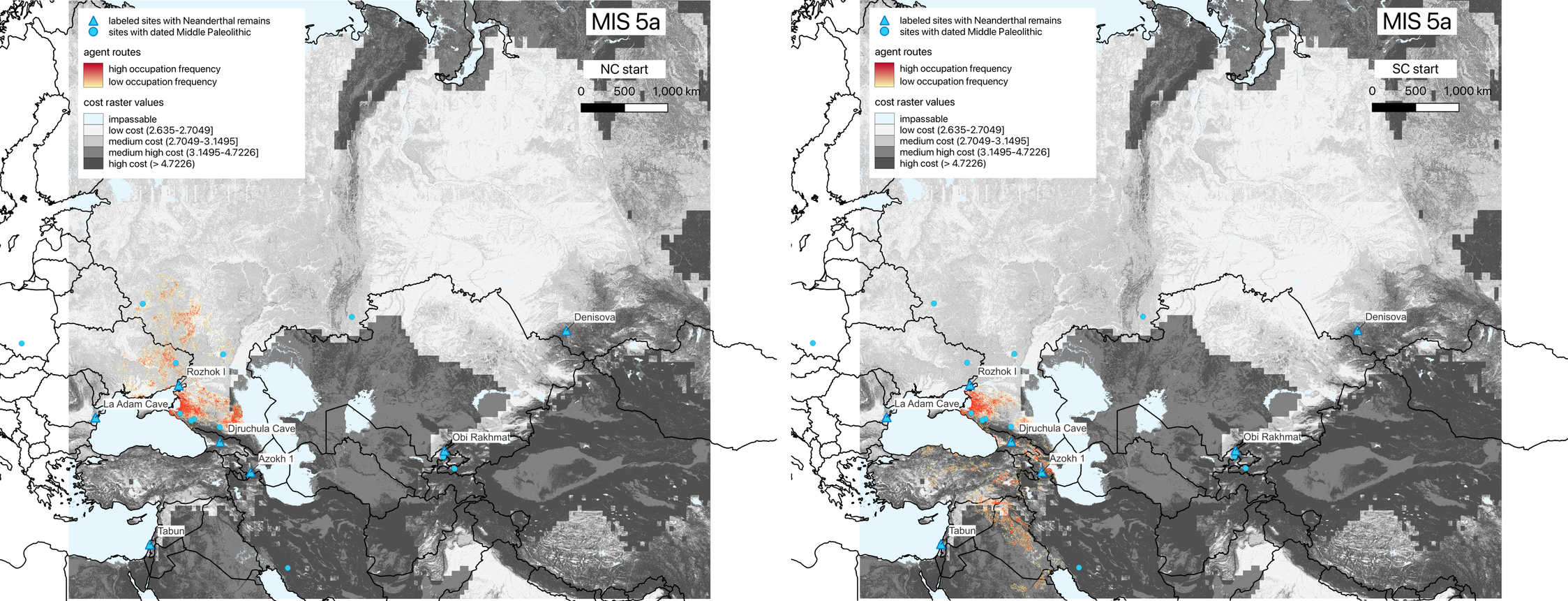


**S7 Fig.** Summary of output paths for all MIS 5a scenarios. Each image shows the frequency of agent steps within cost raster grid squares: locations visited more frequently are shown in red and those visited less frequently are shown in yellow. The base map shows the input cost raster where red is high cost and green is low cost. The left image shows output paths from a Northern Caucasus starting position. The right image shows output paths from a Southern Caucasus starting position.


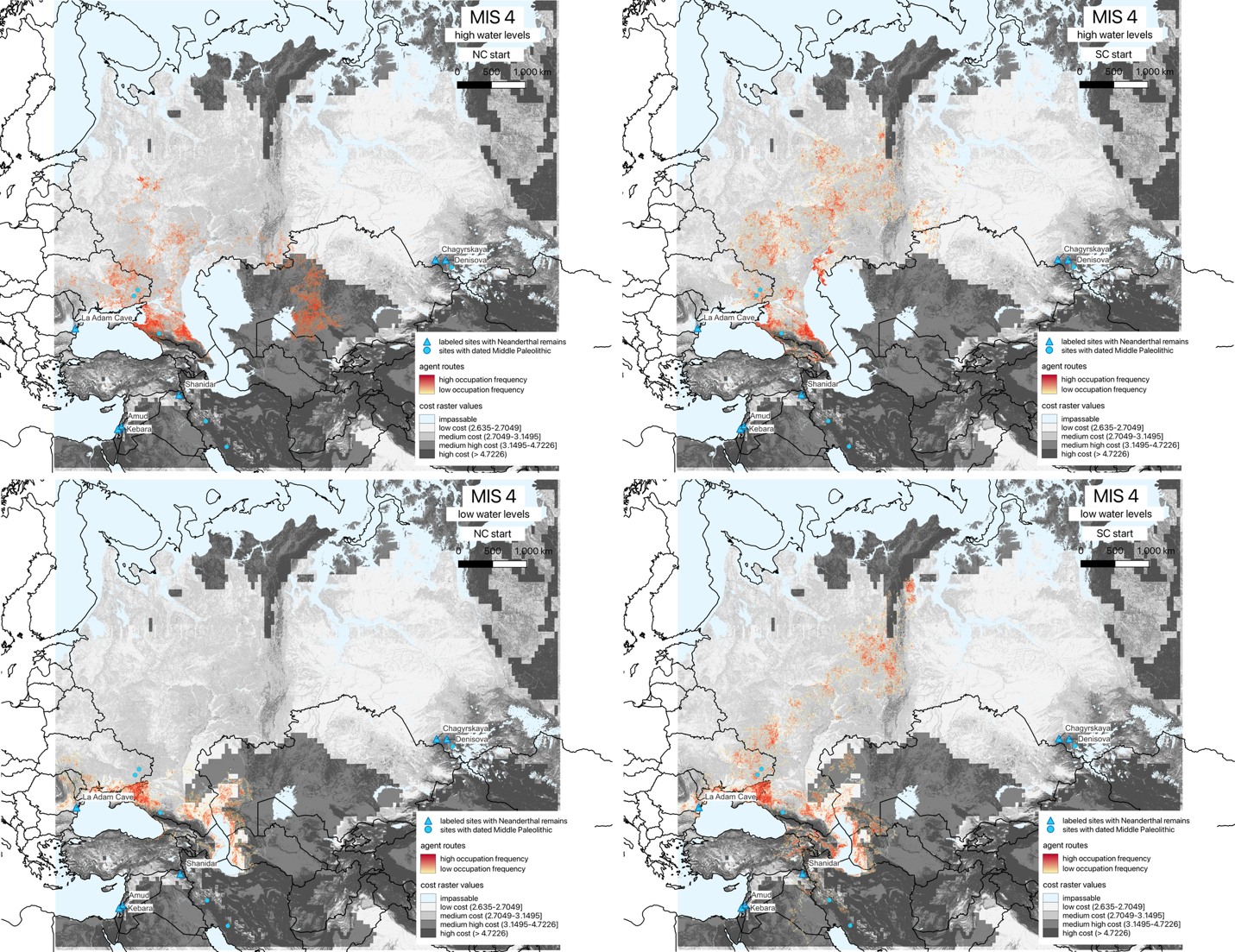


**S8 Fig.** Summary of output paths for all MIS 5d scenarios. Each image shows the frequency of agent steps within cost raster grid squares: locations visited more frequently are shown in red and those visited less frequently are shown in yellow. The base map shows the input cost raster where red is high cost and green is low cost. Cost rasters with high water levels in the left images. Cost rasters with low water levels in the right images. The top two images show output paths from a Northern Caucasus starting position. The bottom two images show output paths from a Southern Caucasus starting position.


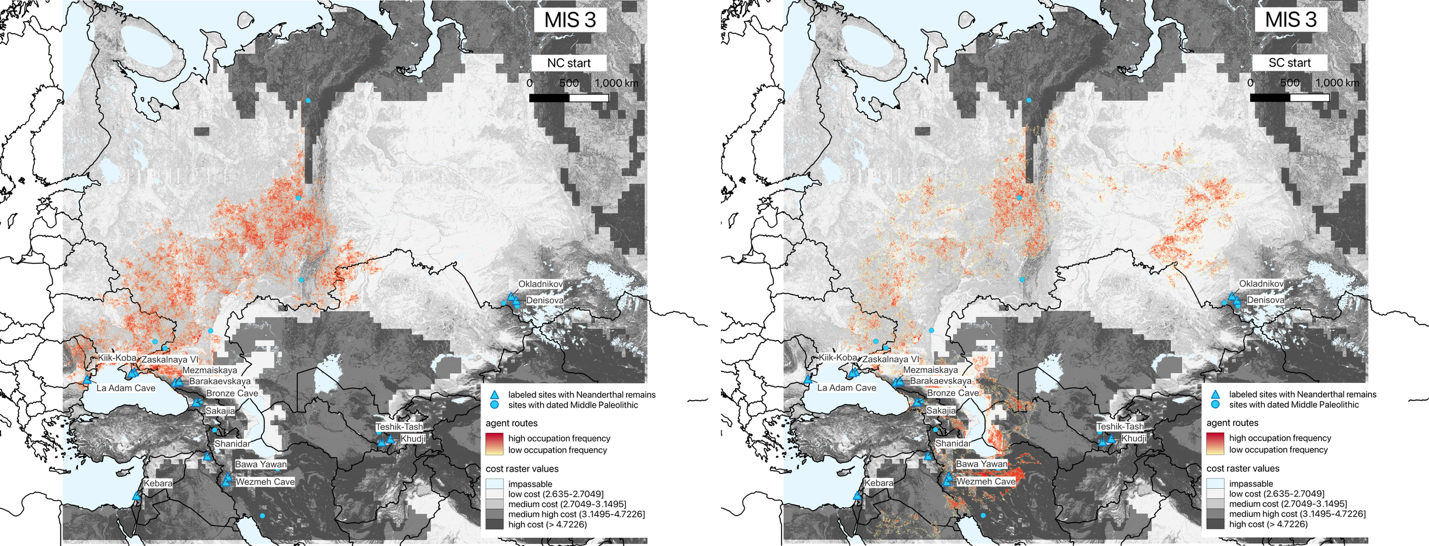


**S9 Fig.** Summary of output paths for all MIS 3 scenarios. Each image shows the frequency of agent steps within cost raster grid squares: locations visited more frequently are shown in red and those visited less frequently are shown in yellow. The base map shows the input cost raster where red is high cost and green is low cost. The left image shows output paths from a Northern Caucasus starting position. The right image shows output paths from a Southern Caucasus starting position.


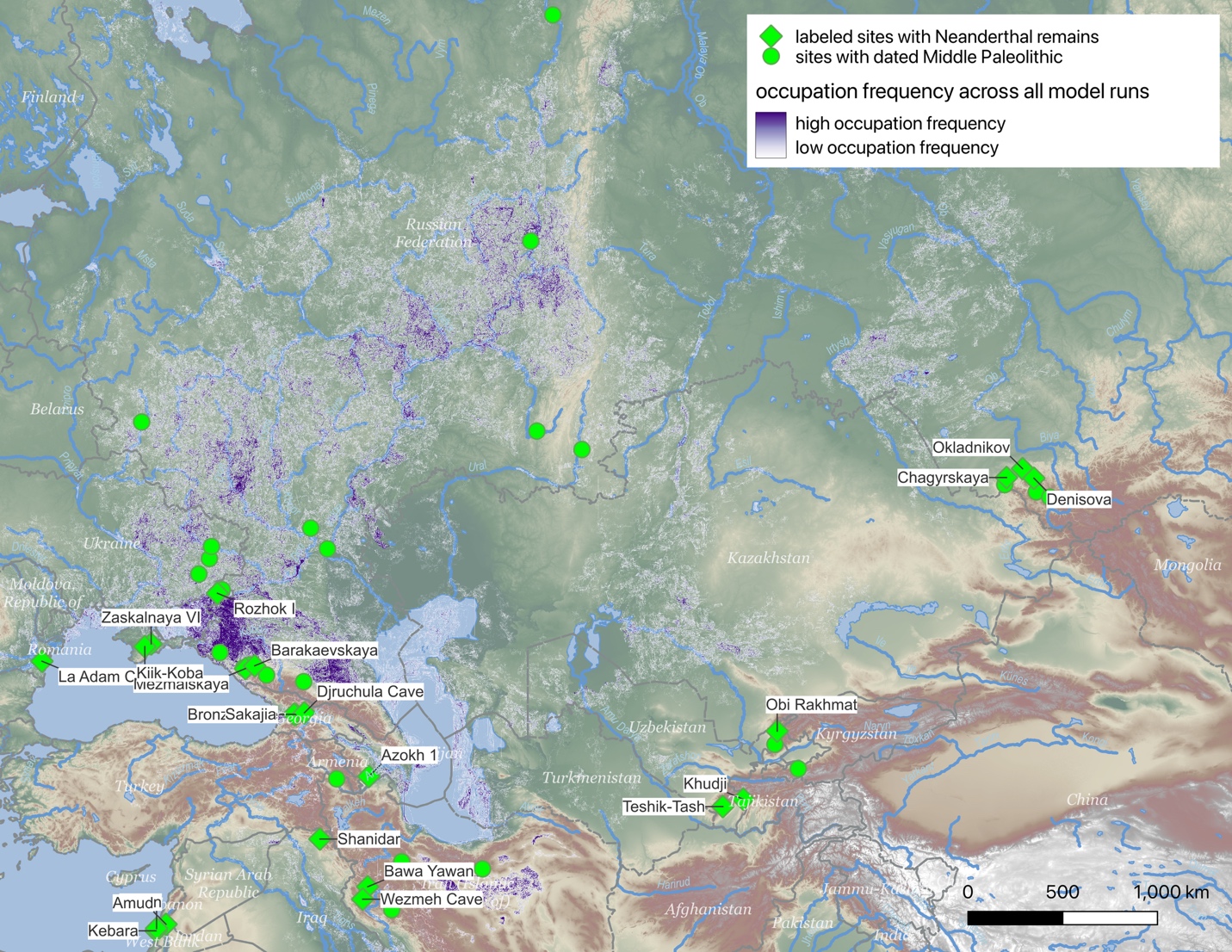


**S10 Fig.** Areas of redundant agent movement with most frequently traversed areas indicated in dark purple and less frequently traversed areas in white. Middle Paleolithic sites (green circles) and sites with Neanderthal remains (green diamonds) dated between MIS 6 and MIS 3 are shown. Base map is derived from a digital elevation model (SRTM15+ V2.5.5) provided by Open Topography [69] with hydrological features from Natural Earth.

## References

1. Grimm V, Berger U, Bastiansen F, Eliassen S, Ginot V, Giske J, et al. A standard protocol for describing individual-based and agent-based models. Ecol Model. 2006;198: 115–126. doi:10.1016/j.ecolmodel.2006.04.023

2. Grimm V, Berger U, DeAngelis DL, Polhill JG, Giske J, Railsback SF. The ODD protocol: A review and first update. Ecol Model. 2010;221: 2760–2768. doi:10.1016/j.ecolmodel.2010.08.019

3. Grimm V, Railsback SF, Vincenot CE, Berger U, Gallagher C, Deangelis DL, et al. The ODD protocol for describing agent-based and other simulation models: A second update to improve clarity, replication, and structural realism. J Artif Soc Soc Simul. 2020;23. Available: http://eprints.bournemouth.ac.uk/33918/

4. Davies B, Holdaway S, Fanning PC. Modeling Relationships Between Space, Movement, and Lithic Geometric Attributes. Am Antiq. 2018;83: 444–461. doi:10.1017/aaq.2018.23

5. Zeynalov AA, Anoikin AA, Kulakov SA, Otcherednoy AK, Kurbanov RN. Gazma Cave—A Final Middle Paleolithic Site in Azerbaijan: Paleogeography, Chronology, Archaeology. Archaeol Ethnol Anthropol Eurasia. 2023;51: 40–49.

6. Fernández-Jalvo Y, King T, Yepiskoposyan L, Andrews P. Introduction: Azokh Cave and the Transcaucasian Corridor. In: Fernández-Jalvo Y, King T, Yepiskoposyan L, Andrews P, editors. Azokh Cave and the Transcaucasian Corridor. Cham: Springer International Publishing; 2016. pp. 1–26. doi:10.1007/978-3-319-24924-7_1

7. Fernández-Jalvo Y, Ditchfield P, Grün R, Lees W, Aubert M, Torres T, et al. Appendix: Dating Methods Applied to Azokh Cave Sites. In: Fernández-Jalvo Y, King T, Yepiskoposyan L, Andrews P, editors. Azokh Cave and the Transcaucasian Corridor. Cham: Springer International Publishing; 2016. pp. 321–339. doi:10.1007/978-3-319-24924-7_16

8. Adler DS, Bar-Yosef O, Belfer-Cohen A, Tushabramishvili N, Boaretto E, Mercier N, et al. Dating the demise: Neandertal extinction and the establishment of modern humans in the southern Caucasus. J Hum Evol. 2008;55: 817–833. doi:10.1016/j.jhevol.2008.08.010

9. Pinhasi R, Nioradze M, Tushabramishvili N, Lordkipanidze D, Pleurdeau D, Moncel M-H, et al. New chronology for the Middle Palaeolithic of the southern Caucasus suggests early demise of Neanderthals in this region. J Hum Evol. 2012;63: 770–780. doi:10.1016/j.jhevol.2012.08.004

10. Moncel M-H, Pleurdeau D, Pinhasi R, Yeshurun R, Agapishvili T, Chevalier T, et al. The Middle Palaeolithic Record of Georgia: A Synthesis of the Technological, Economic and Paleoanthropological Aspects. Anthropol 1962-. 2015;53: 93–125.

11. Pleurdeau D, Moncel M-H, Pinhasi R, Yeshurun R, Higham T, Agapishvili T, et al. Bondi Cave and the Middle-Upper Palaeolithic transition in western Georgia (south Caucasus). Quat Sci Rev. 2016;146: 77–98. doi:10.1016/j.quascirev.2016.06.003

12. Adler D, Tushabramishvili N. MIDDLE PALAEOLITHIC PATTERNS OF SETTLEMENT AND SUBSISTENCE IN THE SOUTHERN CAUCASUS. Tubingen: Kerns Verlag; 2004. pp. 91–132. Available: http://paleo.revues.org/docannexe/image/2464/img

13. Mercier N, Valladas H, Meignen L, Joron J-L, Tushabramishvili N, Adler DS, et al. Dating the Early Middle Palaeolithic Laminar Industry from Djruchula Cave, Republic of Georgia. Paléorient. 2010;36: 163–173.

14. Heydari-Guran S, Benazzi S, Talamo S, Ghasidian E, Hariri N, Oxilia G, et al. The discovery of an in situ Neanderthal remain in the Bawa Yawan Rockshelter, West-Central Zagros Mountains, Kermanshah. PLOS ONE. 2021;16: e0253708. doi:10.1371/journal.pone.0253708

15. Vahdati Nasab H, Berillon G, Jamet G, Hashemi M, Jayez M, Khaksar S, et al. The open-air Paleolithic site of Mirak, northern edge of the Iranian Central Desert (Semnan, Iran): Evidence of repeated human occupations during the late Pleistocene. Comptes Rendus Palevol. 2019;18: 465–478.

16. Ghasidian E, Bretzke K, Conard NJ. Excavations at Ghār-e Boof in the Fars Province of Iran and its bearing on models for the evolution of the Upper Palaeolithic in the Zagros Mountains. J Anthropol Archaeol. 2017;47: 33–49. doi:10.1016/j.jaa.2017.03.001

17. Heydari M, Guérin G, Zeidi M, Conard NJ. Bayesian luminescence dating at Ghār-e Boof, Iran, provides a new chronology for Middle and Upper Paleolithic in the southern Zagros. J Hum Evol. 2021;151: 102926. doi:10.1016/j.jhevol.2020.102926

18. Trinkaus E, Biglari F, Mashkour M, Monchot H, Reyss J-L, Rougier H, et al. Late Pleistocene human remains from Wezmeh Cave, western Iran. Am J Phys Anthropol. 2008;135: 371–378. doi:10.1002/ajpa.20753

19. Zanolli C, Biglari F, Mashkour M, Abdi K, Monchot H, Debue K, et al. A Neanderthal from the Central Western Zagros, Iran. Structural reassessment of the Wezmeh 1 maxillary premolar. J Hum Evol. 2019;135: 102643. doi:10.1016/j.jhevol.2019.102643

20. Bazgir B, Baharvandi E, Azimi M. New Chronology for the Middle Palaeolithic Sequence of Kaldar Cave; Insights from the Third Excavation Season. J Archaeol Archaeom. 2022.

21. Vahdati Nasab H, Berillon G, Hashemi SM, Bahain J-J, Sévêque N, Jayez M, et al. Qaleh Kurd Cave (Qazvin, Iran): Oldest Evidence of Middle Pleistocene Hominin Occupations and a Human Deciduous Tooth in the Iranian Central Plateau. J Paleolit Archaeol. 2024;7: 16. doi:10.1007/s41982-024-00180-4

22. Trinkaus E. The Shanidar Neandertals. Academic Press; 1983.

23. Pomeroy E, Bennett P, Hunt CO, Reynolds T, Farr L, Frouin M, et al. New Neanderthal remains associated with the ‘flower burial’ at Shanidar Cave. Antiquity. 2020;94: 11–26. doi:10.15184/aqy.2019.207

24. Hovers E. The Lithic Assemblages of Amud Cave. In: Akazawa T, Aoki K, Bar-Yosef O, editors. Neandertals and Modern Humans in Western Asia. Boston: Kluwer Academic Publishers; 1998. pp. 143–163. doi:10.1007/0-306-47153-1_10

25. Valladas H, Mercier N, Froget L, Hovers E, Joron J-L, Kimbel WH, et al. TL Dates for the Neanderthal Site of the Amud Cave, Israel. J Archaeol Sci. 1999;26: 259–268. doi:10.1006/jasc.1998.0334

26. Schwarcz HP, Buhay WM, Grün R, Valladas H, Tchernov E, Bar-Yosef O, et al. ESR dating of the Neanderthal site, Kebara Cave, Israel. J Archaeol Sci. 1989;16: 653–659. doi:10.1016/0305-4403(89)90029-0

27. Rebollo NR, Weiner S, Brock F, Meignen L, Goldberg P, Belfer-Cohen A, et al. New radiocarbon dating of the transition from the Middle to the Upper Paleolithic in Kebara Cave, Israel. J Archaeol Sci. 2011;38: 2424–2433. doi:10.1016/j.jas.2011.05.010

28. Grün R, Stringer C. Tabun revisited: revised ESR chronology and new ESR and U-series analyses of dental material from Tabun C1. J Hum Evol. 2000;39: 601–612. doi:10.1006/jhev.2000.0443

29. Coppa A, Grün R, Stringer C, Eggins S, Vargiu R. Newly recognized Pleistocene human teeth from Tabun Cave, Israel. J Hum Evol. 2005;49: 301–315. doi:10.1016/j.jhevol.2005.04.005

30. Krivoshapkin A, Viola B, Chargynov T, Krajcarz MT, Krajcarz M, Fedorowicz S, et al. Middle Paleolithic variability in Central Asia: Lithic assemblage of Sel’Ungur cave. Quat Int. 2020;535: 88–103. doi:10.1016/j.quaint.2018.09.051

31. Cyrek K, Sudoł M, Czyżewski Ł, Osipowicz G, Grelowska M. Middle Palaeolithic cultural levels from Middle and Late Pleistocene sediments of Biśnik Cave, Poland. Quat Int. 2014;326–327: 20–63. doi:10.1016/j.quaint.2013.12.014

32. Iovita R, Doboş A, Fitzsimmons KE, Probst M, Hambach U, Robu M, et al. Geoarchaeological prospection in the loess steppe: Preliminary results from the Lower Danube Survey for Paleolithic Sites (LoDanS). Quat Int. 2014;351: 98–114. doi:10.1016/j.quaint.2013.05.018

33. Praslov ND, Kuznetsova LV. Paleoliticheskoe poselenie Sukhaya Mechetka (po materialam raskopok S. N. Zamyatina) [Palaeolithic site of Sukhaya Mechetka (based on materials of S. N. Zamyatin’s excavations)]. Sankt-Peterburg: Institut istorii material’noi kul’tury RAN; 2020.

34. Golovanova LV, Hoffecker JF, Kharitonov VM, Romanova GP. Mezmaiskaya Cave: A Neanderthal Occupation in the Northern Caucasus. Curr Anthropol. 1999;40: 77–86. doi:10.1086/515805

35. Derevianko AP. Pervonachalʹnoe zaselenie chelovekom severnoy, centralʹnoy i sredney Azii [The original peopling of Northern, Central and Western Central Asia]. Shun’kov MV, editor. Novosibirsk: Izdatelʹstvo Instituta archeologi i ėtnografii SO RAN; 2015.

36. Derevianko AP, Shunkov MV, Agadjanian AK, Baryshnikov GF, Malaeva EM, Ulianov V. Paleoenvironment and paleolithic human occupation of Gorny Altai. Subsistence and adaptation in the vicinity of Denisova Cave. Novosibirsk: Institute of Archaeology and Ethnography SB RAS Press; 2003.

37. Lesage C, Postnov AV, Krivoshapkin AI, Jaubert J. Levallois reduction sequences in Altai: A view from the study of Ust’-Kanskaya Cave (Gorny-Altai, Russia). Quat Int. 2020;535: 104–116. doi:10.1016/j.quaint.2018.09.047

38. Derevianko AP, Markin SV. Mustʹe Gornogo Altai͡a (po materialam peshchery im. Okladnikova) [The Altai Mousterian (based on the materials from Okladnikov Cave]. Novosibirsk: Nauka; 1992.

39. Faerman M, Zilberman U, Smith P, Kharitonov V, Batsevitz V. A Neanderthal infant from the Barakai Cave, Western Caucasus. J Hum Evol. 1994;27: 405–415. doi:10.1006/jhev.1994.1056

40. Golovanova LV, Doronichev VB. The Middle Paleolithic of the Caucasus. J World Prehistory. 2003;17: 71–140. doi:10.1023/A:1023960217881

41. Praslov ND. Rannii paleolit severo-vostochkogo Priazov’ya i nizhnego Dona. Moscow: Nauka; 1968.

42. Pavlov PYu. The Paleolithic of Northeastern Europe: New Data. Archaeol Ethnol Anthropol Eurasia. 2008;33: 33–45. doi:10.1016/j.aeae.2008.04.014

43. Svendsen JI, Heggen HP, Hufthammer AK, Mangerud J, Pavlov P, Roebroeks W. Geo-archaeological investigations of Palaeolithic sites along the Ural Mountains – On the northern presence of humans during the last Ice Age. Quat Sci Rev. 2010;29: 3138–3156. doi:10.1016/j.quascirev.2010.06.043

44. Jacobs Z, Li B, Shunkov MV, Kozlikin MB, Bolikhovskaya NS, Agadjanian AK, et al. Timing of archaic hominin occupation of Denisova Cave in southern Siberia. Nature. 2019;565: 594–599. doi:10.1038/s41586-018-0843-2

45. Kotov VG, Rumyantsev MM, Gimranov DO. Stoyanka srednego paleolita v peshchere Imanai-1 na Yuzhnom Urale: predvaritel’nye itogi arkheologicheskikh issledovaniy [Middle Paleolithic Site of Imanai-1 Cave in the Southern Urals: Preliminary Results of Archaeological Investigations]. Orient Stud. 2020;13: 1271–1291. doi:10.22162/2619-0990-2020-51-5-1271-1291

46. Slimak L, Svendsen JI, Mangerud J, Plisson H, Heggen HP, Brugère A, et al. Late Mousterian Persistence near the Arctic Circle. 2011;332: 6.

47. Blackwell BAB, Liang S, Golovanova LV, Doronichev VB, Skinner AR, Blickstein JIB. ESR at Treugol’naya Cave, Northern Caucasus Mt., Russia: Dating Russia’s oldest archaeological site and paleoclimatic change in Oxygen Isotope Stage 11. Appl Radiat Isot. 2005;62: 237–245. doi:10.1016/j.apradiso.2004.08.005

48. Derevianko AP, Markin SV, Zykin VS, Zykina VS, Zazhigin VS, Sizikova AO, et al. Chagyrskai͡a peshchera-stoi͡anka srednego paleolita Altai͡a [Chagyrskay Cave - a Middle Palaeolithic site in the Altai]. Arkheologii͡a Ėtnografii͡a Antropol Evrazii. 2013; 2–27.

49. Slavinsky VS, Rybin EP, Belousova NE. VARIATION IN MIDDLE AND UPPER PALEOLITHIC TECHNIQUES OF LITHIC REDUCTION AT KARA-BOM, THE ALTAI MOUNTAINS: REFITTING STUDIES. Archaeol Ethnol Anthropol Eurasia. 2016;44: 39–50. doi:10.17746/1563-0110.2016.44.1.039-050

50. Hoffecker JF, Holliday VT, Nehoroshev P, Vishnyatsky L, Otcherednoy A, Salnaya N, et al. The Dating of a Middle Paleolithic Blade Industry in Southern Russia and Its Relationship to the Initial Upper Paleolithic. J Paleolit Archaeol. 2019;2: 381–417. doi:10.1007/s41982-019-00032-6

51. Otcherednoi A, Voskresenskaya E, Stepanova K, Vishnyatsky L, Nekhoroshev P, Larionova A, et al. Complex geoarhaeological studies of the Middle Paleolithic sites in the Russian Plain. Trans Inst Hist Mater Cult Russ Acad Sci. 2018; 74–83. doi:10.31600/2310-6557-2018-17-74-83

52. Hoffecker J, Southon J, Nehoroshev P, Otcherednoy A, Vishnyatsky L. Radiocarbon dating of Rozhok I. 2020.

53. Shchelinskiĭ VE. Ob Ilʹskoĭ mustʹerskoĭ stoi͡anke [On the Mousterian site of Il’skaya]. Strat Plus. 2012; 69–110.

54. Doronicheva E, Golovanova LV, Doronichev VB, Nedomolkin AG, Spasovskiy YuN, Kulkova MA, et al. Nouveau site du Paléolithique Moyen dans le Caucase Central (résultats préliminaires des études complexes). L’Anthropologie. 2020;124: 102758. doi:10.1016/j.anthro.2020.102758

55. Doronicheva EV, Nedomolkin AG, Muriy AA, Kulkova MA, Sapelko TV, Nosevich ES, et al. Hadjoh-2: A Middle Paleolithic Workshop-Camp in Northwestern Caucasus. Archaeol Ethnol Anthropol Eurasia. 2018;46: 16–26. doi:10.17746/1563-0102.2018.46.1.016-026

56. Shirokov VN, Volkov RB, Kosintsev PA, Lapteva EG. Paleoliticheskaya stoyanka Bogdanovka (Yuzhnyi Ural) [The Paleolithic site of Bogdanovka (Southern Ural)]. Rosiyskaya Arkheologiya. 2011; 111–125.

57. Trinkaus E, Ranov VA, Lauklin S. Middle Paleolithic human deciduous incisor from Khudji, Tajikistan. J Hum Evol. 2000;38: 575–584. doi:10.1006/jhev.1999.0370

58. Fagernäs Z, Troché G, Buylaert J-P, Khujageldiev T, Kurbanov R, Olsen JV, et al. Cleaning the Dead: Optimized decontamination enhances palaeoproteomic analyses of a Pleistocene hominin tooth from Khudji, Tajikistan. bioRxiv; 2024. p. 2024.06.13.598810. doi:10.1101/2024.06.13.598810

59. Stepanchuk VN, Vasilyev SV, Khaldeeva NI, Kharlamova NV, Borutskaya SB. The last Neanderthals of Eastern Europe: Micoquian layers IIIa and III of the site of Zaskalnaya VI (Kolosovskaya), anthropological records and context. Quat Int. 2017;428: 132–150. doi:10.1016/j.quaint.2015.11.042

60. Stepanchuk V, Sapozhnikov IV. The Middle and Upper Pleistocene of Ukraine: A Synopsis of Palaeolithic Finds with Special Reference to Patterns of Peopling and Cultural Development. Arheol Mold. 2010;33: 13–24.

61. Trinkaus E, Maley B, Buzhilova AP. Brief communication: Paleopathology of the Kiik-Koba 1 Neandertal. Am J Phys Anthropol. 2008;137: 106–112. doi:10.1002/ajpa.20833

62. Majkić A, d’Errico F, Stepanchuk V. Assessing the significance of Palaeolithic engraved cortexes. A case study from the Mousterian site of Kiik-Koba, Crimea. PLOS ONE. 2018;13: e0195049. doi:10.1371/journal.pone.0195049

63. Glantz M, Viola B, Wrinn P, Chikisheva T, Derevianko A, Krivoshapkin A, et al. New hominin remains from Uzbekistan. J Hum Evol. 2008;55: 223–237. doi:10.1016/j.jhevol.2007.12.007

64. Krause J, Orlando L, Serre D, Viola B, Prüfer K, Richards MP, et al. Neanderthals in central Asia and Siberia. Nature. 2007;449: 902–904. doi:10.1038/nature06193

65. Nishiaki Y, Aripdjanov O. A new look at the Middle Paleolithic lithic industry of the Teshik-Tash Cave, Uzbekistan, West Central Asia. Quat Int. 2021;596: 22–37. doi:10.1016/j.quaint.2020.11.035

66. Krivoshapkin A, Kuzmin YV, Jull AJT. Chronology of the Obi-Rakhmat Grotto (Uzbekistan): First Results on the Dating and Problems of the Paleolithic Key Site in Central Asia. Radiocarbon. 2010;52: 549–554. doi:10.1017/S0033822200045586

67. Kolobova KA, Krivoshapkin AI, Pavlenok KK, Flas D, Derevianko AP, Islamov UI. The Denticulate Mousterian as a supposedly distinct facies in Western Central Asia. Archaeol Ethnol Anthropol Eurasia. 2012;40: 11–23. doi:10.1016/j.aeae.2012.05.003

68. Pavlenok KK, Pavlenok GD, Kogai SA, Khuzhanazarov M. Regional Sources of the Obirakhmatian: New Data from Kulbulak Site. Bull Irkutsk State Univ «Geoarchaeology Ethnol Anthropol Series». 2018; 3–22.

69. Tozer B, Sandwell DT, Smith WHF, Olson C, Beale JR, Wessel P. Global Bathymetry and Topography at 15 Arc Sec: SRTM15+. Earth Space Sci. 2019;6: 1847–1864. doi:10.1029/2019EA000658

70. Leonardi M, Hallett EY, Beyer R, Krapp M, Manica A. pastclim: an R package to easily access and use paleoclimatic reconstructions. bioRxiv; 2022. p. 2022.05.18.492456. doi:10.1101/2022.05.18.492456

71. Batchelor CL, Margold M, Krapp M, Murton DK, Dalton AS, Gibbard PL, et al. The configuration of Northern Hemisphere ice sheets through the Quaternary. Nat Commun. 2019;10: 3713. doi:10.1038/s41467-019-11601-2

72. Messager ML, Lehner B, Grill G, Nedeva I, Schmitt O. Estimating the volume and age of water stored in global lakes using a geo-statistical approach. Nat Commun. 2016;7: 13603. doi:10.1038/ncomms13603

73. Lehner B, Verdin K, Jarvis A. HydroSHEDS technical documentation, version 1.0. World Wildl Fund US Wash DC. 2006; 1–27.

74. Flanders Marine Institute. IHO Sea Areas, version 3. 2018. Available: https://www.marineregions.org/

75. Herzog I. Spatial Analysis Based On Cost Functions. 1st ed. In: Gillings M, Hacıgüzeller P, Lock G, editors. Archaeological Spatial Analysis. 1st ed. Routledge; 2020. pp. 333–358. doi:10.4324/9781351243858-18

76. Herzog I. Theory and practice of cost functions. In: Contreras F, Melero, editors. Fusion of Cultures Proceedings of the 38th Annual Conference on Computer Applications and Quantitative Methods in Archaeology. Granada, Spain; 2013. pp. 375–382.

77. Llobera M, Sluckin TJ. Zigzagging: Theoretical insights on climbing strategies. J Theor Biol. 2007;249: 206–217. doi:10.1016/j.jtbi.2007.07.020

78. Li, Vanwezer N, Boivin N, Gao X, Ott F, Petraglia M, et al. Heading north: Late Pleistocene environments and human dispersals in central and eastern Asia. PLOS ONE. 2019;14: e0216433. doi:10.1371/journal.pone.0216433

79. Field JS, Lahr MM. Assessment of the Southern Dispersal: GIS-Based Analyses of Potential Routes at Oxygen Isotopic Stage 4. J World Prehistory. 2005;19: 1–45. doi:10.1007/s10963-005-9000-6

80. Field JS, Petraglia MD, Lahr MM. The southern dispersal hypothesis and the South Asian archaeological record: Examination of dispersal routes through GIS analysis. J Anthropol Archaeol. 2007;26: 88–108. doi:10.1016/j.jaa.2006.06.001

81. Canosa-Betés J. Border surveillance: Testing the territorial control of the Andalusian defense network in center-south Iberia through GIS. J Archaeol Sci Rep. 2016;9: 416–426. doi:10.1016/j.jasrep.2016.08.026

82. Fábrega-Álvarez P, Fonte J, García FJG. Mobilidade e materialidade: uma aproximação à análise da loca- lização das estátuas-menir transfronteiriças (Norte de Portugal e Sul da Galiza). Estelas e Estátuas-menires: da Pré à Proto-história. Sabugal, Portugal: Centro de Estudos de Arqueologia, Artes e Ciências do Património; 2011. pp. 245–270.

83. Güimil-Fariña A, Parcero-Oubiña C. “Dotting the joins”: a non-reconstructive use of Least Cost Paths to approach ancient roads. The case of the Roman roads in the NW Iberian Peninsula. J Archaeol Sci. 2015;54: 31–44. doi:10.1016/j.jas.2014.11.030

84. Moody JA, Troutman BM. Characterization of the spatial variability of channel morphology. Earth Surf Process Landf. 2002;27: 1251–1266. doi:10.1002/esp.403

85. Frasson RP de M, Pavelsky TM, Fonstad MA, Durand MT, Allen GH, Schumann G, et al. Global Relationships Between River Width, Slope, Catchment Area, Meander Wavelength, Sinuosity, and Discharge. Geophys Res Lett. 2019;46: 3252–3262. doi:10.1029/2019GL082027

86. Arkhipov SA, Ehlers J, Johnson RG, Wright J. Glacial drainage towards the Mediterranean during the Middle and Late Pleistocene. Boreas. 1995;24: 196–206. doi:10.1111/j.1502-3885.1995.tb00773.x

87. Mangerud J. Ice-dammed lakes and rerouting of the drainage of northern Eurasia during the Last Glaciation. Quat Sci Rev. 2004;23: 1313–1332. doi:10.1016/j.quascirev.2003.12.009

88. Astakhov VI. Evidence of Late Pleistocene ice-dammed lakes in West Siberia. Boreas. 2006;35: 607–621. doi:https://doi.org/10.1111/j.1502-3885.2006.tb01167.x

89. Komatsu G, Baker VR, Arzhannikov SG, Gallagher R, Arzhannikova AV, Murana A, et al. Catastrophic flooding, palaeolakes, and late Quaternary drainage reorganization in northern Eurasia. Int Geol Rev. 2016;58: 1693–1722. doi:10.1080/00206814.2015.1048314

90. Zolnikov ID, Postnov AV, Anoikin AA, Glushkova NV, Bychkov DA, Vybornov AV. O paleogeograficheskikh predposylkakh i vozmozhnykh puti͡akh migrat͡sii paleoliticheskogo cheloveka s zapadnykh predgoriĭ Urala v dolinu nizhneĭ Obi [Paleogeographical Conditions and Possible Migration Routes of Paleolithic Humans from the Western Foothills of the Urals to the Valley of the Lower Ob River]. Probl Archaeol Ethnogr Anthropol Sib Neighboring Territ. 2019;25: 109–115. doi:10.17746/2658-6193.2019.25.109-115

91. Panin AV, Astakhov VI, Lotsari E, Komatsu G, Lang J, Winsemann J. Middle and Late Quaternary glacial lake-outburst floods, drainage diversions and reorganization of fluvial systems in northwestern Eurasia. Earth-Sci Rev. 2020;201: 103069. doi:10.1016/j.earscirev.2019.103069

92. Létolle R, Micklin P, Aladin N, Plotnikov I. Uzboy and the Aral regressions: A hydrological approach. Quat Int. 2007;173–174: 125–136. doi:10.1016/j.quaint.2007.03.003

93. Conrad O, Bechtel B, Bock M, Dietrich H, Fischer E, Gerlitz L, et al. System for Automated Geoscientific Analyses (SAGA) v. 2.1.4. Geosci Model Dev. 2015;8: 1991–2007. doi:10.5194/gmd-8-1991-2015

94. Svitoch AA. The history of the last Aral Sea. Arid Ecosyst. 2009;15: 5–17.

95. Kurbanov R, Murray A, Thompson W, Svistunov M, Taratunina N, Yanina T. First reliable chronology for the Early Khvalynian Caspian Sea transgression in the Lower Volga River valley. Boreas. 2021;50: 134–146. doi:https://doi.org/10.1111/bor.12478

96. Dolukhanov PM, Chepalyga AL, Lavrentiev NV. The Khvalynian transgressions and early human settlement in the Caspian basin. Quat Int. 2010;225: 152–159. doi:10.1016/j.quaint.2009.10.039

97. Gavrilov A, Pavlov V, Fridenberg A, Boldyrev M, Khilimonyuk V, Pizhankova E, et al. The current state and 125&thinsp;kyr history of permafrost on the Kara Sea shelf: modeling constraints. The Cryosphere. 2020;14: 1857–1873. doi:10.5194/tc-14-1857-2020

98. Svitoch AA, Selivanov AO, Yanina TA. Paleohydrology of the Black Sea Pleistocene Basins. Water Resour. 2000;27: 594–603.

99. Bezrodnykh Y, Yanina T, Sorokin V, Romanyuk B. The Northern Caspian Sea: Consequences of climate change for level fluctuations during the Holocene. Quat Int. 2020;540: 68–77. doi:10.1016/j.quaint.2019.01.041

100. Larsen E, KJæR KH, Demidov IN, Funder S, Grøsfjeld K, Houmark-Nielsen M, et al. Late Pleistocene glacial and lake history of northwestern Russia. Boreas. 2006;35: 394–424. doi:10.1080/03009480600781958

101. Dalton AS, Gowan EJ, Mangerud J, Möller P, Lunkka JP, Astakhov V. Last interglacial (MIS 5e) sea level proxies in the glaciated Northern Hemisphere. Geosciences – Palaeooceanography, Palaeoclimatology; 2021 Nov. doi:10.5194/essd-2021-367

102. Mangerud J, Astakhov V, Jakobsson M, Svendsen JI. Huge Ice-age lakes in Russia. J Quat Sci. 2001;16: 773–777. doi:https://doi.org/10.1002/jqs.661

103. Korsakova OP. Pleistocene marine deposits in the coastal areas of Kola Peninsula (Russia). Quat Int. 2009;206: 3–15. doi:10.1016/j.quaint.2008.11.004

104. Költringer C, Stevens T, Bradák B, Almqvist B, Kurbanov R, Snowball I, et al. Enviromagnetic study of Late Quaternary environmental evolution in Lower Volga loess sequences, Russia. Quat Res. 2021;103: 49–73. doi:10.1017/qua.2020.73

105. Yanina TA. Correlation of the Late Pleistocene paleogeographical events of the Caspian Sea and Russian Plain. Quat Int. 2012;271: 120–129. doi:10.1016/j.quaint.2012.06.003

106. Binford LR. Constructing Frames of Reference: An Analytical Method for Archaeological Theory Building Using Hunter-Gatherer and Environmental Data Sets. Berkeley, CA: University of California Press; 2001.

107. Marwick B, Johnson A, White D, Eff EA. binford: Binford’s Hunter-Gatherer Data. 2016. Available: https://cran.r-project.org/web/packages/binford/index.html

108. McLean DJ, Skowron Volponi MA. trajr: An R package for characterisation of animal trajectories. Ethology. 2018;124: 440–448. doi:10.1111/eth.12739
